# Mitochondrial uncoupling protein 1 antagonizes atherosclerosis by blocking NLRP3 inflammasome–dependent interleukin-1β production

**DOI:** 10.1126/sciadv.abl4024

**Published:** 2021-12-08

**Authors:** Ping Gu, Xiaoyan Hui, Qiantao Zheng, Yuan Gao, Leigang Jin, Weimin Jiang, Changsheng Zhou, Tianxia Liu, Yu Huang, Qing Liu, Tao Nie, Yanfang Wang, Yu Wang, Jianguo Zhao, Aimin Xu

**Affiliations:** 1 State Key Laboratory of Pharmaceutical Biotechnology, University of Hong Kong, Hong Kong, China.; 2 Department of Medicine, University of Hong Kong, Hong Kong, China.; 3 Department of Endocrinology, Jinling Hospital, Nanjing University, School of Medicine, Nanjing, China.; 4 School of Biomedical Sciences, Chinese University of Hong Kong, Hong Kong, China.; 5 State Key Laboratory of Stem Cell and Reproductive Biology, Chinese Academy of Sciences, Chaoyang District, Beijing, China.; 6 Savaid Medical School, University of Chinese Academy of Sciences, Beijing, China.; 7 Department of Cardiology, The Affiliated Hospital of Nanjing University of Chinese Medicine, Jiangsu Province Hospital of Traditional Chinese Medicine, Nanjing, China.; 8 Department of Medical Imaging, Jinling Hospital, Medical School of Nanjing University, Nanjing, China.; 9 Department of Biomedical Sciences, City University of Hong Kong, Hong Kong, China.; 10 Clinical Department of Guangdong Metabolic Disease Research Center of Integrated Chinese and Western Medicine, The First Affiliated Hospital of Guangdong Pharmaceutical University, Guangzhou, China.; 11 State Key Laboratory of Animal Nutrition, Institute of Animal Science, Chinese Academy of Agricultural Sciences, Beijing, China.; 12 Department of Pharmacy and Pharmacology, University of Hong Kong, Hong Kong, China.

## Abstract

Mitochondrial uncoupling protein 1 (UCP1) is the hallmark of brown adipocytes responsible for cold- and diet-induced thermogenesis. Here, we report a previously unidentified role of UCP1 in maintaining vascular health through its anti-inflammatory actions possibly in perivascular adipose tissue. UCP1 deficiency exacerbates dietary obesity-induced endothelial dysfunction, vascular inflammation, and atherogenesis in mice, which was not rectified by reconstitution of UCP1 in interscapular brown adipose tissue. Mechanistically, lack of UCP1 augments mitochondrial membrane potential and mitochondrial superoxide, leading to hyperactivation of the NLRP3-inflammasome and caspase-1–mediated maturation of interleukin-1β (IL-1β). UCP1 deficiency–evoked deterioration of vascular dysfunction and atherogenesis is reversed by IL-1β neutralization or a chemical mitochondrial uncoupler. Furthermore, UCP1 knockin pigs (which lack endogenous UCP1) are refractory to vascular inflammation and coronary atherosclerosis. Thus, UCP1 acts as a gatekeeper to prevent NLRP3 inflammasome activation and IL-1β production in the vasculature, thereby conferring a protective effect against cardiovascular diseases.

## Teaser

The mitochondrial protein UCP1 has potent therapeutic effects on vascular inflammation and atherosclerosis.

## INTRODUCTION

Atherosclerosis, a chronic inflammatory disease characterized by the accumulation of fatty deposits along the innermost layer of the arteries, is the primary cause of cardiovascular diseases (CVDs). Endothelial dysfunction and vascular inflammation underlie all phases in the development of atherosclerosis (*1*, *2*). A large body of experimental and epidemiological studies have unequivocally established that obesity-associated adipose dysfunction is among the major culprits that cause endothelial dysfunction, vascular inflammation, and atherosclerotic plaque development (*3*).

Most blood vessels, except the cerebral vasculature, are surrounded by perivascular adipose tissue (PVAT) (*4*). In humans, local expansion of PVAT is closely associated with hypertension, vascular calcification, and development of atherosclerosis upon obesity (*5*). PVAT expansion and inflammation precede endothelial dysfunction and atherosclerotic plaque formation in hypercholesterolemic mouse and pig models (*6*–*9*). Transplantation of PVAT, but not visceral or subcutaneous adipose from obese mice, induces endothelial dysfunction and augments atherosclerosis in mice (*10*, *11*). These findings support the notion that inflamed PVAT is an important contributor to vascular inflammation, endothelial dysfunction, and CVDs in obesity. However, how PVAT inflammation is orchestrated remains obscure.

PVAT, especially those surrounding the thoracic aorta, is phenotypically brown (*12*). Positron emission tomography–based scans detect a considerable amount of active brown adipose tissue (BAT) around the aortic region in adult human (*13*, *14*). The gene expression pattern of thoracic PAVT is almost identical to interscapular BAT (iBAT) in mice, and PVAT from human coronary artery also expresses brown adipocyte–specific genes such as uncoupling protein 1 (UCP1) (*15*). The browning level of PVAT is inversely associated with immune cell infiltration, proinflammatory cytokine/adipokine production, hypertension, and atherosclerosis progression (*16*–*18*). Notably, induction of adipose tissue browning by cold exposure alleviates atherosclerosis and improves endothelial function in mice with intact PVAT, but this effect is absent in PVAT-deficient mice (*12*), demonstrating that the abolishment in the browning feature of PVAT is causatively linked to pathogenesis of CVDs (*16*).

To elucidate the mechanism underlying PVAT browning–evoked protection against CVDs, we investigated the role of UCP1 on obesity/diabetes-induced endothelial dysfunction, vascular inflammation, and atherosclerosis in both mice and pigs. As a hallmark of brown adipocytes, UCP1 is best known for its role in adaptive thermogenesis by uncoupling the proton gradient [also referred to as mitochondrial membrane potential (MMP)] with ATP (adenosine 5′-triphosphate) synthesis (*19*). Here, we uncovered an unexpected anti-inflammatory and antiatherosclerotic role of UCP1 by blocking mitochondrial superoxide (mtSuperoxide)–induced activation of the NLR family pyrin domain containing 3 (NLRP3) inflammasome and production of interleukin-1β (IL-1β) possibly in PVAT. Furthermore, we demonstrated that pharmacological interventions targeting the UCP1/MMP/NLRP3 inflammasome–IL-1β axis are promising therapeutic strategies for the treatment of atherosclerosis in small- and large-animal models.

## RESULTS

### UCP1 deficiency exacerbates obesity-evoked vascular dysfunction and atherosclerosis

**Fig. 1.**
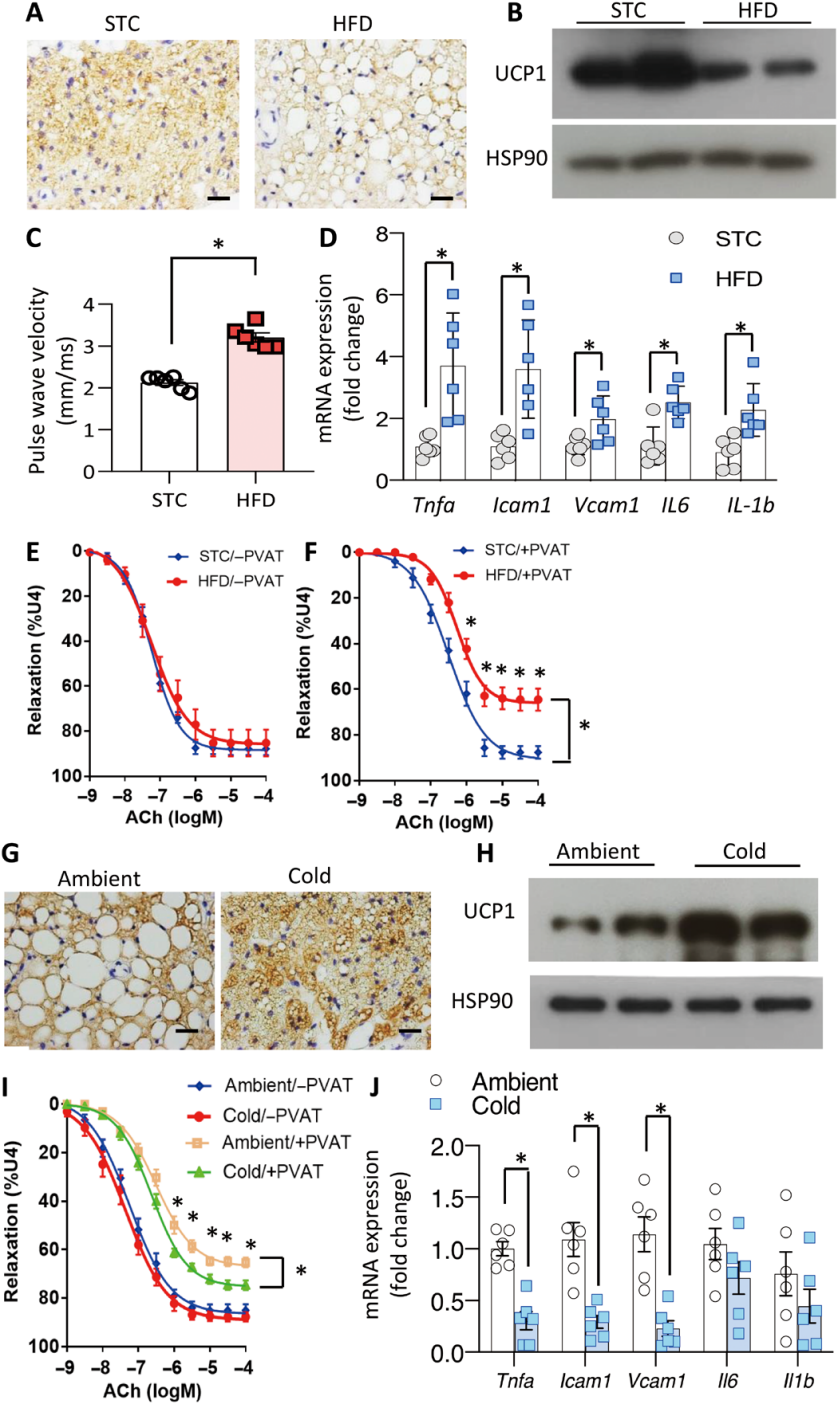
Obesity- and cold temperature–induced changes in vascular function is closely related to alterations in UCP1 expression in PVAT. (**A** to **F**) Six-week-old male C57BL/6J mice were fed with standard chow (STC) or high-fat diet (HFD) for 12 weeks before aorta and PVAT were isolated for analysis. (A) Immunohistochemical staining of UCP1 in PVAT. Scale bars, 100 μm. (B) Western blot analysis of UCP1 in PVAT. (C) Pulse wave velocity in thoracic aorta of the mice was measured by ultrasonography. *n* = 6, *P < 0.05 versus STC mice. (D) Real-time polymerase chain reaction (PCR) analysis for proinflammatory genes in mouse aorta, normalized to rps18, *n* = 6, *P < 0.05 versus STC mice. (E and F) Acetylcholine (ACh)–induced vasorelaxation in aortic rings without (E) or with (F) PVAT, assessed using wire myographs. *n* = 7 to 8, *P < 0.05 versus STC mice. (**G** to **J**), C57BL/6J mice on HFD were housed at cold (6°C) or ambient temperature (24°C) for 6 days before analysis. (G) Immunohistochemical staining of UCP1 in PVAT. Scale bars, 100 μm. (H) Western blot of UCP1 in PVAT. (I) ACh-induced vasorelaxation in aortic rings with or without PVAT. *n* = 13 to 15, *P < 0.05 versus 24°C. (J) The mRNA expression of proinflammatory genes in mouse aorta. *n* = 6, *P < 0.05 versus 24°C.

Compared to that in lean mice, expression of UCP1 within PVAT was notably reduced in obese mice (Fig. 1, A and B), accompanied by potentiated arterial stiffness and expression of inflammatory genes in the blood vessel wall of the thoracic aorta (Fig. 1, C and D). Furthermore, the aortic rings from obese mice exhibited remarkably impaired acetylcholine (ACh)–evoked vasorelaxation compared to the lean controls when the surrounding PVAT remained unremoved (Fig. 1F and table S1), whereas the difference disappeared in the absence of PVAT (Fig. 1E), indicating that dietary obesity-induced endothelial dysfunction is attributed to dysfunctional PVAT. Conversely, cold exposure of obese mice markedly induced UCP1 expression in PVAT (Fig. 1, G and H), which was associated with a more potent antirelaxation activity by PVAT (Fig. 1I and table S2), down-regulation of inflammatory markers and adhesion molecules in aorta (Fig. 1J).

*Ucp1* knockout (KO) mice were used to define the role of UCP1 in regulation of vascular function. Despite similar body weight, fat mass, lipid profiles, and glucose tolerance (fig. S1), *Ucp1* KO mice displayed more severe vascular inflammation upon diet-induced obesity compared to the wild-type (WT) littermates, as evidenced by higher expression of proinflammatory cytokines in thoracic aortas (fig. S2A). In aortas with PVAT, endothelium-dependent vasodilation was significantly impaired in obese *Ucp1* KO mice compared with WT mice (fig. S2C and table S3). Notably, no difference on vasorelaxation was observed between WT and KO mice when PVAT was removed (fig. S2B), suggesting that obesity-induced endothelial dysfunction is at least partially mediated by PVAT and is further exacerbated by UCP1 deficiency within PVAT. The artery stiffness was also elevated in obese KO mice (fig. S2D). Furthermore, compared with apoE^−/−^ mice, *Ucp1/apoE* double KO (DKO) mice displayed more prominent atherosclerotic plaques in the ascending aortas and in the aortic roots, although they had comparable body weight, fat mass, glucose tolerance, and lipid profiles (figs. S2, E to M, and S3).

At room temperature, UCP1 is predominantly expressed in PVAT and iBAT. We therefore reconstituted the UCP1 expression in iBAT of DKO mice by direct injection of adenovirus-associated viruses (AAVs) expressing mouse UCP1 or green fluorescent protein (GFP) into iBAT (fig. S4, A and B). Despite a comparable level of UCP1 between apoE^−/−^ and DKO mice with forced expression of UCP1 in iBAT, the atherosclerotic plaque development and vascular inflammation were not improved in DKO mice with reconstitution of UCP in iBAT, compared with the DKO littermates receiving AAVs expressing GFP (fig. S4, C to G), suggesting that the vascular protection of UCP1 is not attributed to its actions in classical iBAT and is possibly through the action of UCP1 in PVAT.

### UCP1 deficiency enhances activation of the NLRP3 inflammasome–IL-1β axis

**Fig. 2.**
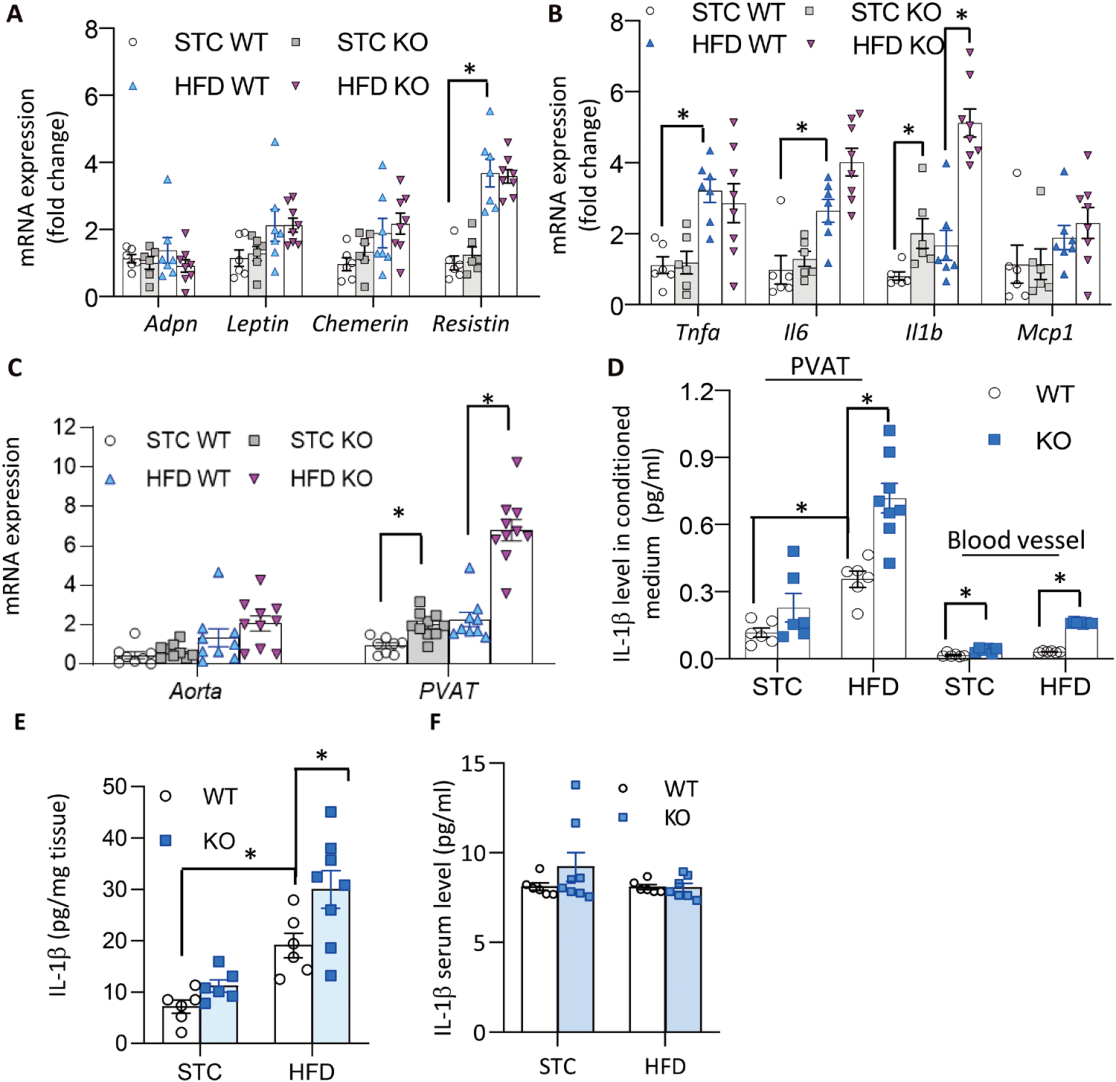
IL-1β expression is increased in UCP1-deficient PVAT. WT and *Ucp1* KO mice were fed with STC or HFD for 12 weeks before isolation of aorta and surrounding PVAT. (**A** and **B**) Expression levels of mRNA encoding several adipokines (A) and inflammatory cytokines (B) in PVAT, determined by real-time PCR analysis, *n* = 6 to 8. (**C**) Comparison of mRNA expression of Il-1β in aortas and PVAT, *n* = 8 to 10. (**D**) IL-1β protein levels in the conditional medium from ex vivo culture of aorta and PVAT, measured by enzyme-linked immunosorbent assay (ELISA), *n* = 6 to 8. (**E**) IL-1β protein in PVAT lysates and (**F**) in serum determined by ELISA, *n* = 6 to 8. *P < 0.05.

To interrogate how loss of UCP1 in PVAT accelerates vascular inflammation, expression levels of those genes potentially involved in modulating vascular function were examined. Compared to WT mice, the adipokines, including adiponectin, leptin, chemerin, resistin, and visfatin, remained largely unchanged in the PVAT of *Ucp1* KO mice (Fig. 2A). The levels of inflammatory cytokines, especially IL-1β (*Il1b*), were substantially higher in PVAT of obese KO mice than in WT counterparts (Fig. 2B), and the mRNA expression of *Il1b* was present in a much higher level in PVAT than in blood vessel wall (Fig. 2C). To further compare the relative level of IL-1β in the blood vessel wall and PVAT, equal length of the aorta was dissected and isolated into PVAT and blood vessel fractions followed by collection of the conditioned medium and the tissue lysates. Within the equal length of the aorta, the level of IL-1β protein secreted from the PVAT fraction was much higher than that from nude blood vessel in both lean and obese mice (~8-fold; Fig. 2D). Notably, UCP1 deficiency enhanced IL-1β secretion from PVAT but had little effect on blood vessels. Consistently, IL-1β level in the PVAT lysates of obese *Ucp1* KO mice was significantly higher than that in obese WT mice (Fig. 2E). In contrast, serum levels of IL-1β were comparable among all the mice strains examined (Fig. 2F), suggesting that UCP1 is involved in the vascular, but not systemic, IL-1β production.

### IL-1β mediates UCP1 deficiency–induced endothelial dysfunction and atherosclerosis in mice

**Fig. 3.**
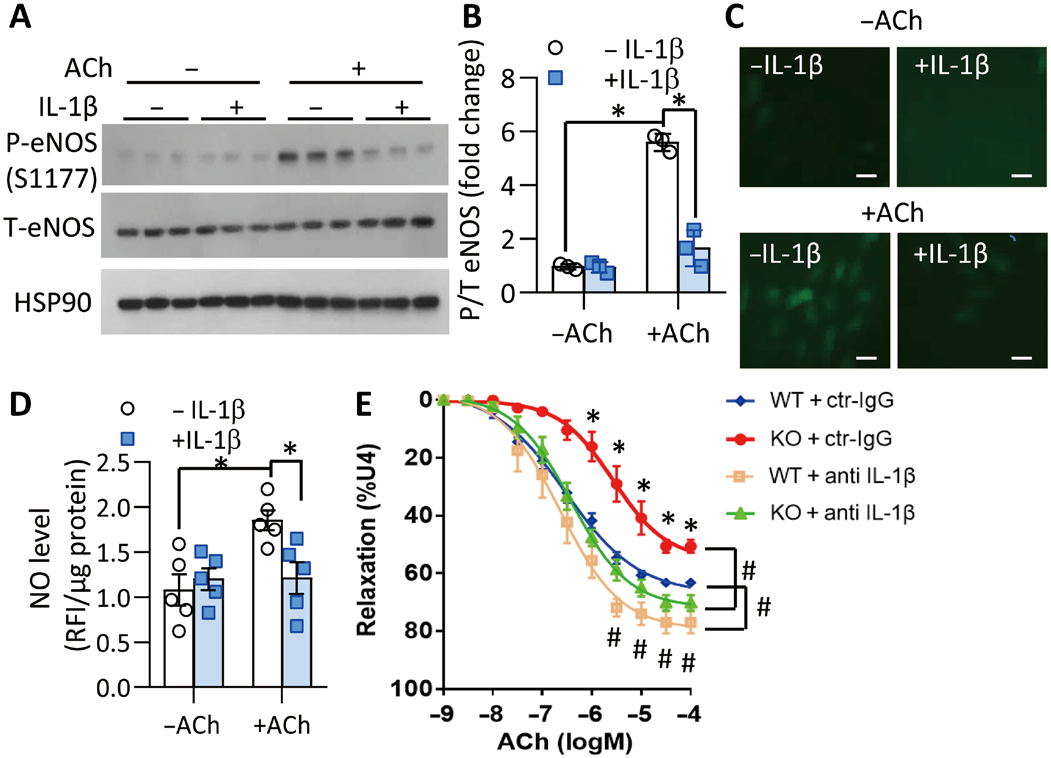
Neutralization of IL-1β improves endothelial dysfunction. (**A** to **D**) Human aortic endothelial cells were stimulated with ACh (10 μM) in the absence or presence of recombinant IL-1β (10 ng/ml). (A) Western blotting analysis for phosphorylated eNOS on S1177, total eNOS, and HSP90 (as a loading control). (B) Densitometric quantification for the ratio of phospho/total eNOS in (A). *n* = 3. *P < 0.05. (C and D) Cell permeable fluorescent NO probe 4,5-diaminofluorescein diacetate (DAF-2DA) was added to the cells to measure intracellular NO production. (C) Representative fluorescence images of DAF-2DA staining to visualize NO production (scale bars, 20 μm). (D) Intracellular NO levels quantified by reading relative fluorescence intensity (RFI) of DAF-2DA with a microplate reader and normalized to protein concentration. *n* = 5, *P < 0.05. (**E**) The aortic rings with PVAT from WT and *Ucp1* KO mice on HFD for 12 weeks were subjected to measurement of ACh-evoked vasorelaxation, after incubation with an IL-1β–neutralizing antibody or isotype-matched control immunoglobulin G (IgG; 2 μg/ml) for 1 hour. *n* = 6 to 7, *P < 0.05 versus WT, #*P* < 0.05 versus control IgG.

The direct effects of IL-1β on endothelial nitric oxide synthase (eNOS) activation and nitric oxide (NO) production were first tested in human aortic endothelial cells (HAoEC). ACh-induced eNOS phosphorylation (Ser^1177^) was largely abolished by addition of recombinant IL-1β, accompanied with a reduction in NO production (Fig. 3, A and D). When aortic rings with PVAT were incubated with a neutralizing antibody against mouse IL-1β (*20*), ACh-evoked vasorelaxation was improved in both WT and *Ucp1* KO mice, and the difference between these mice became indistinguishable (Fig. 3E and table S4). Furthermore, daily administration of Anakinra, the recombinant human IL-1 receptor antagonist (*21*) (fig. S5), decreased arterial stiffness and the atherosclerotic plaques of DKO mice to a level similar to that of apoE^−/−^ mice (fig. S6, A to H). These findings suggest that elevated IL-1β in UCP1-deficient PVAT at least in part contributes to vascular pathologies in UCP1-deficient mice.

### Increased MMP links UCP1 deficiency to hyperactivation of NLRP3 inflammasome and overproduction of mtSuperoxide

**Fig. 4.**
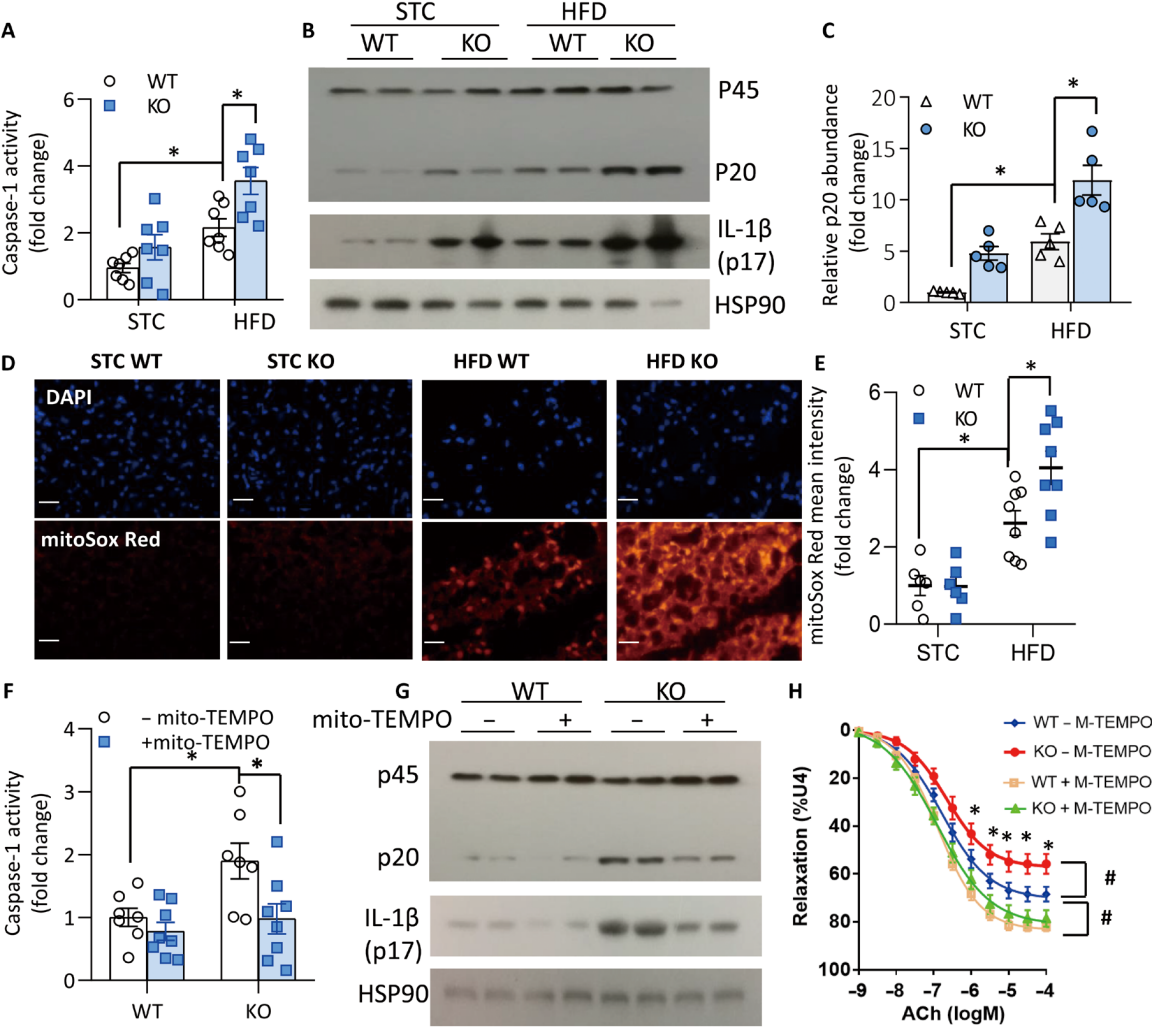
UCP1 deficiency exacerbates obesity-induced activation of caspase-1 and overproduction of mitochondria superoxide. (**A** to **E**) WT and *Ucp1* KO mice were fed with STC or HFD for 12 weeks, and aortic PVAT was collected for further analysis. (A) Caspase-1 activity in PVAT measured by the colorimetric assay and normalized with protein concentration. *n* = 7. *P < 0.05. (B) Western blotting for pro- (p45) and mature (p20) caspase-1 and IL-1β in PVAT. (C) Densitometric quantification of p20 in (B). *n* = 5. *P < 0.05. (D) Representative images of PVAT stained with 4′,6-diamidino-2-phenylindole (DAPI) or mitoSox Red to visualize nuclei and mtSuperoxide. (E) Quantification of fluorescence intensities of mitoSox Red staining images in (D) by Image J software. Scale bars, 20 μm. *n* = 6 to 8. *P < 0.05. (**F** and **G**) PVATs from WT and *Ucp1* KO mice were cultured in Dulbecco’s modified Eagle’s medium (DMEM)/F12 for 16 hours in the presence or absence of Mito-TEMPO. (F) Caspase-1 activity and (G) Western blotting for caspase-1 cleavage and IL-1β in PVAT, *n* = 7 to 8. *P < 0.05. (**H**) ACh-induced vasorelaxation in aortic rings of obese WT and *Ucp1* KO mice pretreated with or without mito-TEMPO for 10 min, in the presence of PVAT. *n* = 15. *P < 0.05 versus KO with mito-TEMPO, #*P* < 0.05 versus WT without mito-TEMPO.

Maturation of IL-1β from its precursor pro–IL-1β is catalyzed by caspase-1 in the NLRP3 inflammasome (*22*). The enzymatic activity of caspase-1 in PVAT of obese *Ucp1* KO mice was significantly increased compared with that in obese WT mice (Fig. 4A). Consistently, the cleavage of pro-caspase-1 (p45) into its mature form (p20) and the levels of mature IL-1β in PVAT were elevated upon UCP1 deficiency (Fig. 4, B and C). Likewise, pharmacological inhibition of NLRP3 inflammasome with MCC950 markedly decreased production of mature IL-1β in *Ucp1* KO PVAT to a level comparable to that in WT PVAT (fig. S7, A and B) and also reversed the exacerbating effect of *Ucp1* KO PVAT on impairment of endothelium-dependent vasorelaxation in mouse aortas (fig. S7C and table S5). Furthermore, staining of PVAT sections showed the accumulation of mtSuperoxide, which reportedly triggers activation of the NLRP3 inflammasomes (*23*), was significantly higher in *Ucp1* KO mice than in WT littermates (Fig. 4, D and E). Treatment with the mtSuperoxide chelator mito-TEMPO inhibited caspase-1 activity and IL-1β protein level in PVAT (Fig. 4, F and G). Preincubation of intact aortic rings with mito-TEMPO reversed the impairment on endothelium-dependent vasorelaxation in both obese WT and *Ucp1* KO mice to a comparable level (Fig. 4H and table S6). These data suggest that UCP1 deficiency leads to production of mtSuperoxide, which, in turn, activates the inflammasome–IL-1β axis to induce endothelial dysfunction.

**Fig. 5.**
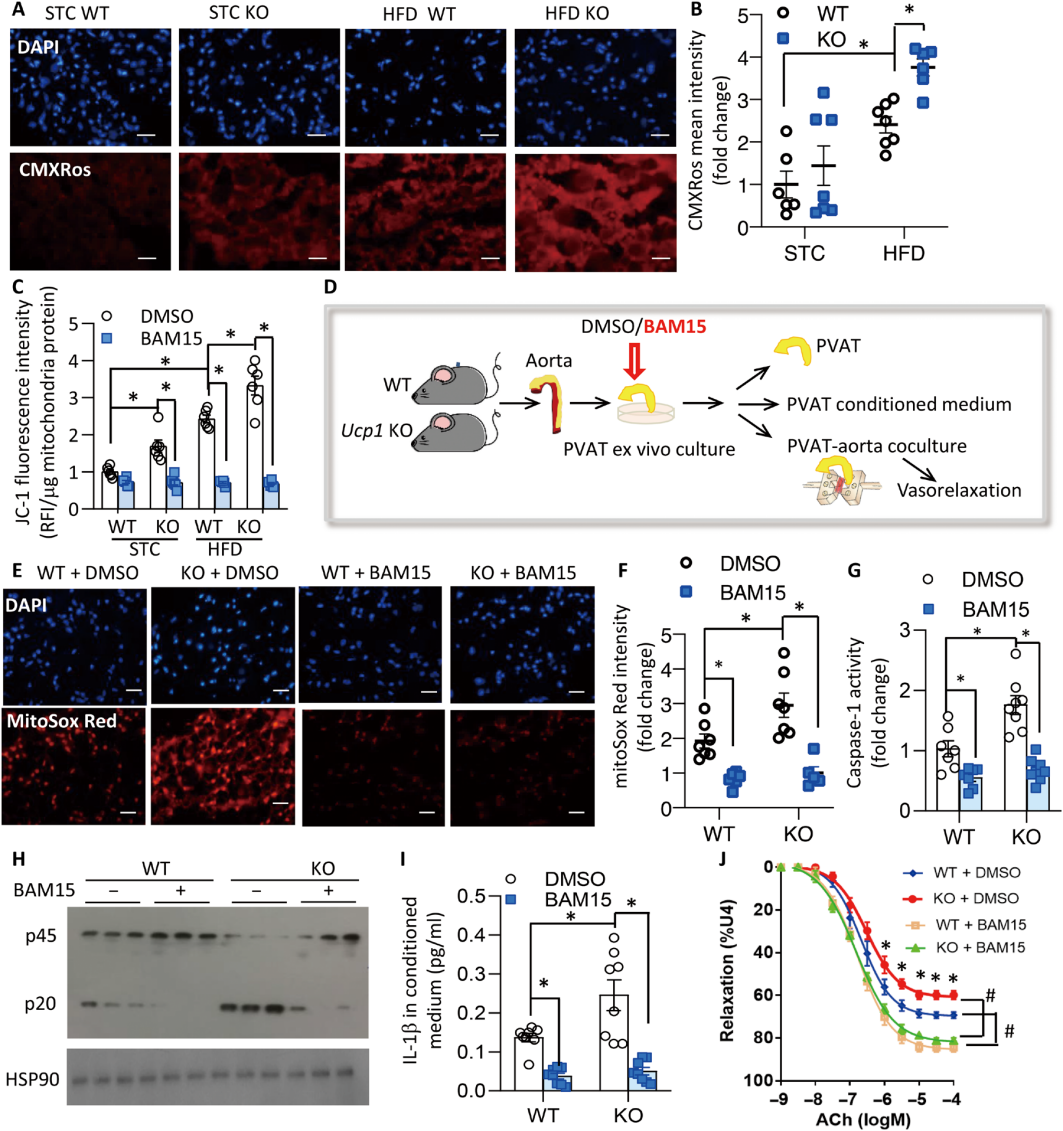
BAM15 alleviates UCP1 deficiency–induced elevation of mitochondrial membrane potential and mtSuperoxide in PVAT. (**A**) Mitochondrial membrane potential (MMP) in PVAT measured by CMXRos staining. Scale bars, 20 μm. (**B**) Quantification of fluorescence intensity of CMXRos staining in (A). *n* = 6 to 7. *P < 0.05. (**C**) MMP in freshly isolated PVAT mitochondria was measured by JC-1 dye. *n* = 6. *P < 0.05. (**D**) Diagram illustrating the ex vivo experimental design in (E) to (J). PVAT was isolated and incubated with BAM15 (1 μM) or dimethyl sulfoxide (DMSO) before PVAT and conditional medium were harvested for further analysis. Alternatively, aortic rings from lean WT mice were cocultured with PVAT pretreated with BAM15, followed by measurement of vasorelaxation with wire myograph. (**E**) mtSuperoxide in PVAT measured by mitoSox Red staining. Scale bars, 20 μm. (**F**) Quantification of fluorescence intensity in (E). *n* = 5 to 7. *P < 0.05. (**G**) Relative caspase-1 activity and (H) Western blotting for caspase-1 cleavage in PVAT, *n* = 7 to 8. *P < 0.05. (**I**) IL-1β concentration in PVAT conditioned medium, *n* = 8. *P < 0.05. (**J**) Vasorelaxation in aortic rings cocultured with PVAT. *n* = 6 to 7, *P < 0.05 versus WT, #*P* < 0.05 versus DMSO.

mtSuperoxide mainly comes from complex I and complex III of the mitochondria electron transport chain during reverse electron transport, a process potentiated by elevated MMP (*24*). Staining with the MMP indicator CMXRos showed that deletion of UCP1 significantly enhanced MMP in PVAT, and the changes were more pronounced in diet-induced obese condition (Fig. 5, A and B). A similar trend in MMP was also observed when mitochondria isolated from PVAT were stained with JC-1 dye (Fig. 5C). Reduction of MMP by the chemical uncoupler BAM15 lowered mtSuperoxide in WT and *Ucp1* KO mice to a comparable level (Fig. 5, D to F, and fig. S8). In accompany, inflammasome activation was suppressed, along with significantly reduced IL-1β in conditioned medium (Fig. 5, G to I). Coculture experiments demonstrated that PVAT of obese *Ucp1* KO mice caused greater impairment on vasodilatation than that from obese WT littermates, whereas this trend was abrogated by pretreatment of PVAT with BAM15 (Fig. 5J and table S7).

Succinate metabolism within brown adipocytes has been implicated in mitochondrial reactive oxygen species (mtROS) production and hepatic inflammation (*25*, *26*). Therefore, we also examined the effect of the succinate dehydrogenase (SDH) inhibitor dimethyl malonate (DMM) on mtSuperoxide production in PVAT. While inhibition of SDH modestly reduced the mtSuperoxide in PVAT of both WT and *Ucp1* KO mice, the significant difference between the two groups remained unchanged (fig. S9), suggesting that succinate oxidation is not a major contributor to UCP1 deficiency–associated mtSuperoxide production in PVAT.

### Mitochondria uncoupling alleviates vascular dysfunction and atherosclerosis in mice

**Fig. 6.**
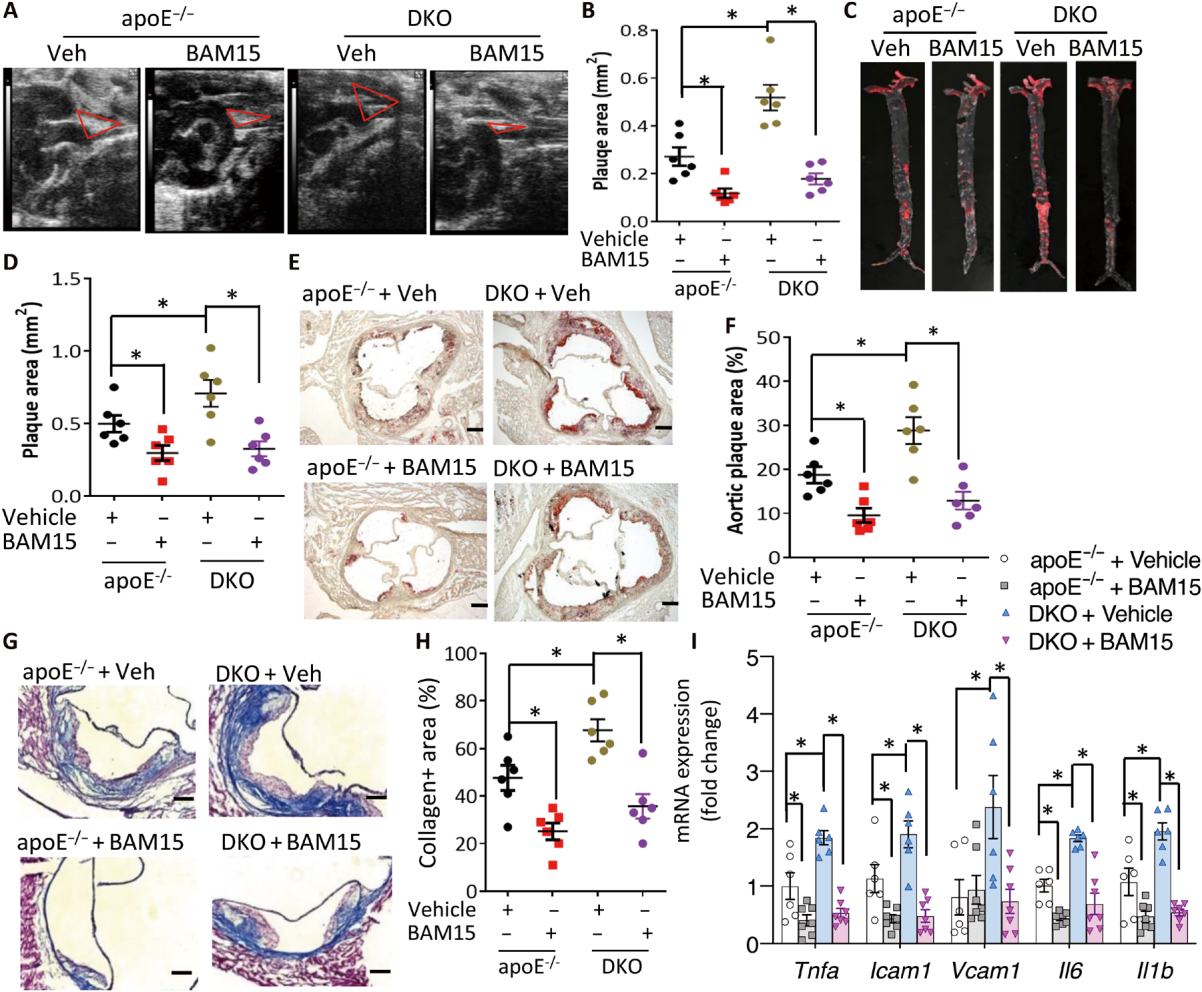
The mitochondrial uncoupler BAM15 counteracts UCP1 deficiency–induced exacerbation of atherosclerosis. DKO and apoE^−/−^ mice on HFHC diet were intraperitoneally administered with BAM15 (0.5 mg kg^−1^ day^−1^) or vehicle control (3% DMSO, 50% PEG400) for 12 weeks. (**A**) Visualization of the brachiocephalic artery branches by ultrasonography and (**B**) quantification of the plaque area, *n* = 6. (**C**) Representative photos of en face oil red staining of entire aorta and (**D**) quantification of the oil red O–stained plaque area. *n* = 6. (**E**) Representative oil red O–stained cross sections of aortic sinus. Scale bars, 200 μm. (**F**) Quantification of the oil red O–stained plaque area in (E), *n* = 6. (**G**) Representative Masson trichrome–stained cross sections of aortic sinus. Scale bars, 100 μm. (**H**) Quantification of collagen-positive area in aortic sinus. *n* = 6. (**I**) Real-time PCR analysis for mRNA expression of several inflammatory genes in aortas, *n* = 6 to 7. *P < 0.05.

To monitor the long-term effect of mitochondrial uncoupling in vivo, apoE^−/−^ and DKO mice were supplemented daily with BAM15 or vehicle solution intraperitoneally for 12 weeks (0.5 mg kg^−1^ day^−1^). Supplementation of BAM15 at this low dosage did not significantly alter the body weight, fat mass, lipid profiles, or circulating markers of inflammation (fig. S10). By contrast, BAM15 treatment substantially reduced the caspase-1 activity in PVAT in both apoE^−/−^ and DKO mice (fig. S11, A to C). Furthermore, BAM15 treatment markedly decreased the atherosclerotic plaque areas to a similar level in the two groups of mice (Fig. 6, A to F) and also reversed the production of UCP1 deficiency–induced production of inflammatory cytokines (fig. S11D). Likewise, exacerbated fibrosis, augmented production of proinflammatory cytokines, macrophage infiltration, and smooth muscle proliferation in the aorta of DKO mice were also attenuated to a level comparable to apoE^−/−^ mice after treatment with BAM15 (Fig. 6, G to I, and fig. S12).

### Reconstitution of functional UCP1 alleviates diabetes/hypercholesteromia-induced vascular dysfunction and atherosclerosis in pigs

**Fig. 7.**
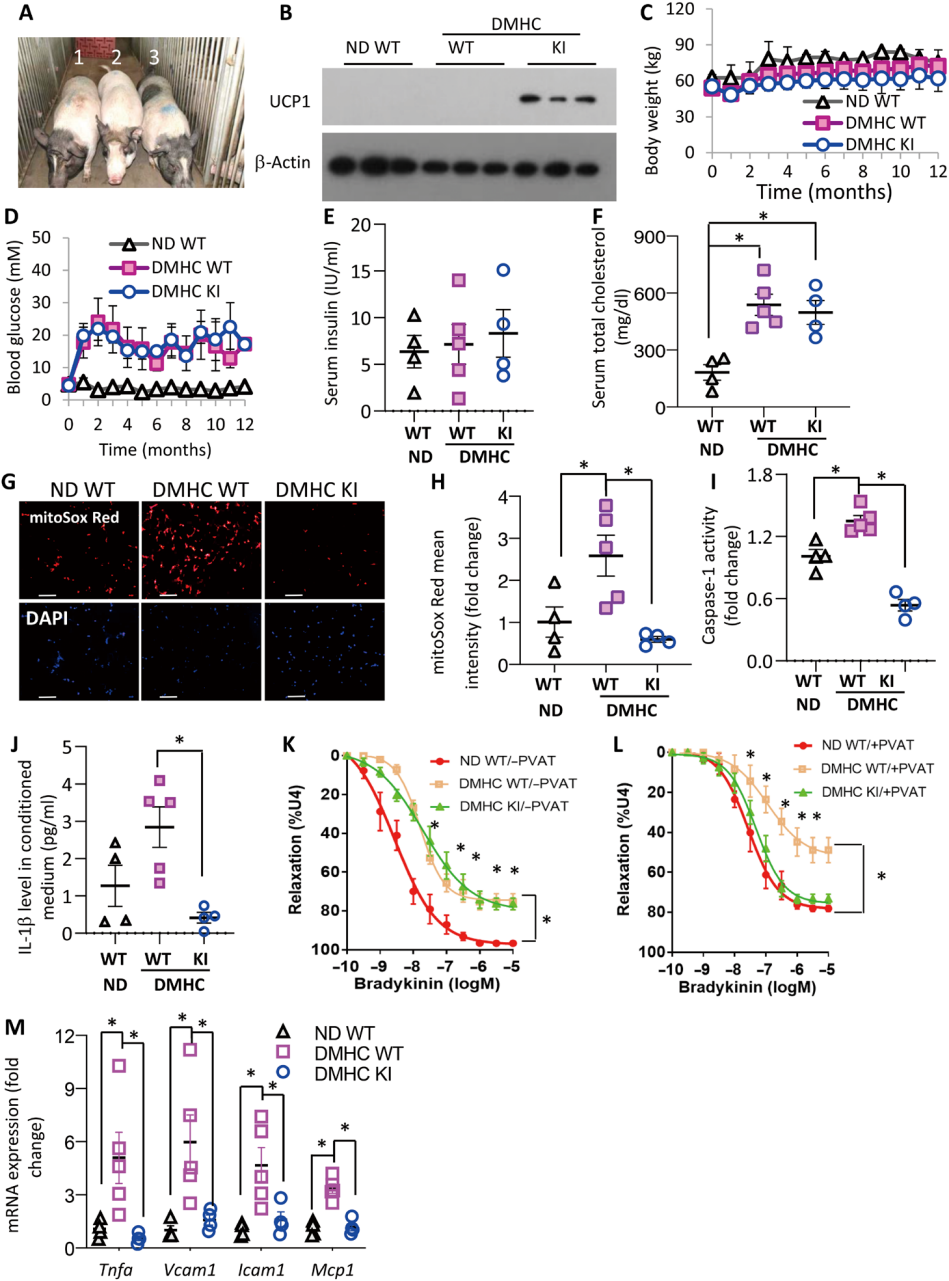
Reconstitution of functional UCP1 dampens diabetes/hypercholesterolemia-induced activation of the mtSuperoxide/caspase-1/IL-1β axis in PVAT and vascular dysfunction in pigs. Male WT and UCP1 KI pigs were injected with streptozotocin to induce diabetes followed by HFHC feeding to induce hypercholesterolemia (DMHC) for 12 months. Age-paired WT pigs fed normal diet (ND) were used as the control. (**A**) Gross appearance of DMHC WT and UCP1 KI pigs at 12 months of age. 1, ND WT; 2, DMHC WT; 3, DMHC KI. *n* = 4 to 5. (**B**) Western blot analysis of UCP1 expression in PVAT of left anterior descending coronary artery (LAD) of the pigs. (**C**) Body weight and (**D**) Blood glucose levels of the pigs measured at different time points after diet induction, *n* = 4 to 5. (**E** and **F**) Plasma insulin (E) and total cholesterol levels (F) measured at 12 months after induction of DMHC, *n* = 4 to 5. *P < 0.05. (**G**) Representative confocal images showing mitoSox staining of PVAT in LAD to measure mtSuperoxide and (**H**) Quantification of relative fluorescence intensity in (G), *n* = 4 to 5. *P < 0.05. (**I**) Caspase-1 activity in PVAT surrounding LADs of the pig, *n* = 4 to 5. *P < 0.05. (**J**) One hundred milligrams of PVAT surrounding pig LADs was cultured in 1 ml of DMEM/F12 ex vivo for 16 hours to collect the conditioned medium. Concentration of IL-1β was measured by ELISA, *n* = 4 to 5. *P < 0.05. (**K** and **L**) Bradykinin-induced vasorelaxation in LAD rings with PVAT (L) and without PVAT (K) by wire myograph, *n* = 6. K, *P < 0.05 versus ND WT; L, *P < 0.05 versus DMHC KI or ND WT. (**M**) Real-time PCR analysis for mRNA expression of proinflammatory genes in LAD, *n* = 4 to 5. *P < 0.05. Photo credit: Qiantao Zheng, Savaid Medical School, University of Chinese Academy of Sciences.

Pigs lack a functional UCP1 gene (*27*). Adipocyte-specific UCP1 knockin (KI) pigs we generated previously (*28*) were used to assess the vascular protective role of UCP1 in large animals. To this end, 6-month-old male adipose-specific UCP1 KI pigs and age-matched WT controls were injected with streptozotocin for 5 days to induce diabetes, followed by feeding with high-fat, high-cholesterol diet (HFHC) to induce vasculopathology (Fig. 7, A and B). Both UCP1 KI pigs and WT controls had similar body weight and developed a comparable degree of diabetes mellitus and hypercholesterolemia (DMHC) after 12 months of HFHC diet (Fig. 7, C to F). However, the level of mtSuperoxide in PVAT surrounding the left anterior descending coronary artery (LAD) was significantly lower in UCP1 KI pigs compared with the WT group with the same treatment (Fig. 7, G and H). Likewise, DMHC-induced elevation of caspase-1 activity in PVAT of UCP1 KI pigs was substantially lower than in WT pigs (Fig. 7I). Furthermore, PVAT of DMHC WT pigs secreted significantly higher amount of IL-1β than their littermates with normal diet, and such an elevation was absent in UCP1 KI pigs (Fig. 7J).

In the presence of PVAT, LAD from WT pigs with DMHC exhibited a significant impairment on bradykinin-evoked (endothelium-dependent) vasodilatation compared with those from WT pigs on normal diet, whereas such DMHC-induced impairments on vasodilation were significantly reduced in UCP1 KI pigs (Fig. 7L and table S8). Notably, the difference in vasodilatation among WT pigs and UCP1 KI pigs with DMHC became indistinguishable when PVAT was removed from LAD (Fig. 7K and table S8), supporting the notion that UCP1 alleviates DMHC-induced endothelial dysfunction through its action on PVAT. Likewise, DMHC-induced expressions of inflammatory cytokines and adhesion molecules in LAD were largely abrogated in UCP1 KI pigs (Fig. 7M).

**Fig. 8.**
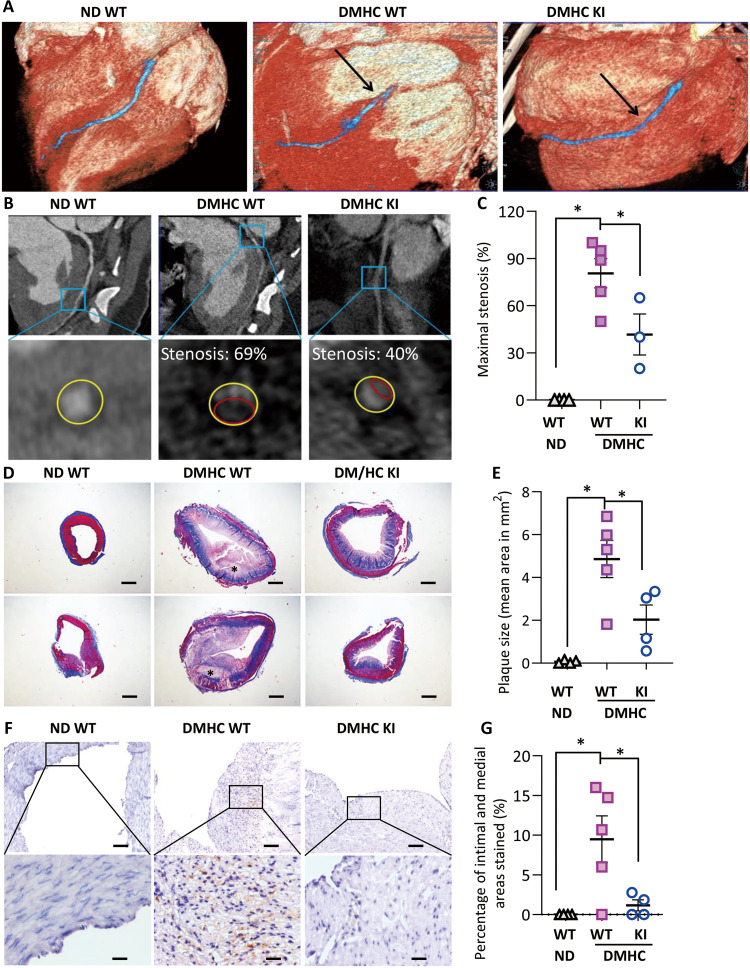
UCP1 KI pigs are protected against DMDH-induced atherosclerosis. WT and UCP1 KI pigs fed with ND or induced to DMHC for 12 months. (**A** to **C**) The pigs were subjected to CT. (A) Volume rendering image showing the LAD after induction of DMHC. Arrows denote the most severe stenosis site. (B) Curved planar reformation (upper) and transverse section of the most severe stenosis section (lower) in LAD. Yellow circle, blood vessel wall; red circle, plaque. (C) Percentage of stenosis in LAD at the site of the most severe stenosis. *n* = 3 to 5, *P < 0.05 versus DMHC WT. (**D**) Photomicrographs of Masson trichrome–stained cross sections in LAD. * denotes necrotic core. Red, smooth muscle; blue, collagen. Scale bars, 500 μm. (**E**) Quantification of the plaque area in pig LAD. *n* = 4 to 5, *P < 0.05 versus DMHC WT. (**F**) Immunohistochemical staining for the macrophage marker cathepsin S in LCA. Scale bars, 100 μm (top) and 20 μm (bottom). (**G**) Quantification of the cathepsin S–stained macrophages in lesion areas. *n* = 4 to 5, *P < 0.05 versus DMHC WT.

In vivo visualization of atherosclerotic plaques in LAD using computed tomography with volume rendering showed that WT pigs on normal diet did not develop atherosclerotic plaques, whereas obvious narrowing in LAD was observed in age-matched DMHC WT pigs (Fig. 8A). Notably, DMHC-induced arterial narrowing in UCP1 KI pigs was much less prominent than in WT pigs (Fig. 8A). Quantification in LAD by curved planar reformation demonstrated that ~80% of the LAD area in DMHC WT pigs developed severe stenosis, which was significantly higher than in DMHC UCP1 KI pigs (~45%) (Fig. 8, B and C).

Atherosclerotic lesions in the UCP1 KI group appeared to be less severe and less complex, with noticeably better preservation of the medial layer, compared with the lesions from the WT group (Fig. 8D). Coronary lesions in the DMHC WT group were more advanced than those in the UCP1 KI pigs according to the modified American Heart Association criteria (*29*, *30*), with four out of the five WT pigs showing a fibrous or thin fibrous cap atheroma, whereas none of the KI pigs developed complex plaques under the same treatment (Fig. 8, D and E). Immunohistochemical staining for the macrophage marker cathepsin S detected the presence of a large number of macrophages in the LAD lesion area of WT pigs with DMHC but was much less severe in lesions of the UCP1 KI pigs (Fig. 8, F and G).

## DISCUSSION

The mitochondrial inner membrane protein UCP1, which is exclusively expressed in beige/brown adipocytes, is traditionally regarded as a thermogenin responsible for dissipation of excessive energy as heat. There is growing interest in developing pharmacological activators of UCP1 as antiobesity agents, despite the fact that UCP1-deficient mice do not exhibit any obesogenic phenotype. In the present study, we identified a previously unidentified function of UCP1 in protection against vascular dysfunction and atherosclerosis, through its inhibition of MMP and mtROS-induced activation of NLRP3 inflammasome in PVAT. Furthermore, we uncovered the loss of browning phenotype in PVAT as an important mediator of obesity-induced vascular inflammation and atherosclerosis by exacerbating the production of IL1β, which in turn acts in a paracrine manner on the adjacent vessel wall.

Atherosclerosis is classically considered as an “inside-out” response of blood vessels that begins with the adhesion of circulatory inflammatory cells to the dysfunctional intimal endothelium, triggering neointimal plaque formation (*31*). A growing body of evidence suggests that dysfunctional PVAT, which is in close proximity to blood vessels with no adventitial fascial boundary, contributes to endothelial dysfunction, vascular inflammation, and atherosclerosis in an “outside-in” manner (*15*, *32*–*35*). Our study demonstrates that obesity-induced endothelial dysfunction in thoracic and coronary arteries is dependent on the presence of PVAT. Furthermore, impairments in endothelium-dependent vasodilation and inflammation in arteries of UCP1-deficient mice are abrogated by removal of PVAT, whereas overexpression of functional UCP1 in pig, or chemical uncoupler to mimic UCP1 activation in PVAT, is sufficient to alleviate endothelial dysfunction. A recent retroprospective study in 134,529 individuals reveals a strong, independent correlation between BAT activity and lower incidence of CVDs (*36*). Our study provides a possible mechanism whereby the brown character of PVAT exerts its cardiovascular-protective functions.

IL-1β is a potent inflammatory cytokine that plays a central role in mediating proinflammatory reactions in response to pathogen-induced tissue injury by binding to its receptor IL-1R (*37*). Aberrant production and/or actions of IL-1β have been implicated in the pathogenesis of a myriad of inflammatory diseases. IL-1β is causally involved in almost every step of the atherogenic process (*38*). The atheroma formation in ApoE^−/−^ mice is decreased by genetic depletion of IL-1β (*39*) or IL-1R (*40*), administration of recombinant IL-1Ra (*41*), or neutralizing antibody against IL-1β (*42*). Likewise, atherosclerotic burden in LDLR^−/−^ mice is reduced by overexpression of IL-1Ra but is exacerbated by IL-1Ra deficiency (*43*). The CANTOS (Canakinumab Anti-Inflammatory Thrombosis Outcome Study) clinical trial, which shows that treatment with canakinumab leads to a notable reduction in recurrent cardiovascular events in patients previously diagnosed with CVD, provides compelling proof for the IL-1β atherothrobosis concept (*44*). Notably, the benefit of canakinumab on cardiovascular events is independent of lipid profiles and blood pressure but is closely related to reduction in inflammatory responses, further reinforcing the effectiveness for IL-1β–based therapy in prevention of CVD. Mechanistically, IL-1β exerts its adverse effects on several types of vascular cells to induce endothelial activation, proliferation/migration of smooth muscle cells, and production of procoagulant, as well as other proinflammatory cytokines (*45*–*47*).

Despite its well-established proatherogenic role, the cellular sources for augmented IL-1β in vasculature remains unresolved. IL-1β is abundantly present in human atherosclerotic plaques (*48*), but its circulating level is below detection even in patients with CVDs (*44*), suggesting that IL-1β is predominantly produced and acts locally. In this connection, our present study identifies PVAT as a major source for vascular IL-1β, which functions in a paracrine manner on adjacent blood vessels to foster vascular inflammation and atherosclerosis.

It should also be noted that Dong *et al.* (*49*) reported a decrease in atherosclerogenesis in UCP1-deficient mice upon cold stress, which was attributed by a notable reduction in cold-induced lipolysis and blood cholesterol. The mice in our study were housed at ambient temperature with conceivably low lipolysis, and no notable difference in serum lipid profiles was observed between DKO and apoE^−/−^ mice. Nevertheless, UCP1 deletion exacerbated atherosclerotic plaque development. A recent study showed that the phenotype of UCP1-deficient mice was temperature dependent (*50*). Thus, the seemingly contradictory observations imply that UCP1 has dual functions in the pathology of CVDs, i.e., it is either antioxidative or prolipolytic, with each mechanism predominating in thermal or cold condition, respectively.

The mitochondrial chemical uncouplers prevent ROS formation by lowering MMP, a condition that thermodynamically disfavors the reverse transport from complex II to I. Historically, the classical pharmacological uncoupler 2,4-dinitrophenol (DNP) was used to be prescribed as a treatment for obesity, and recently, very low, weight-neutral doses of the chemical uncouplers were reevaluated for their therapeutic benefits for ageing-related chronic diseases (*51*). Low dose of DNP improves functional outcomes in animal models of Alzheimer’s and Parkinson’s diseases, epilepsy, and cerebral ischemic stroke (*52*). A liver-targeted DNP analog safely decreases obesity-related insulin resistance, hypertriglyceridemia, nonalcoholic fatty liver, steatohepatitis, and diabetes in high fat–fed rats (*53*, *54*). In our study, long-term low-dose administration of BAM15, a mitochondrial protonophore with more specific action on MMP and less toxicity (*55*), confers protection against obesity-induced vascular inflammation and atherosclerosis by blocking the mtSuperoxide/NLRP3 inflammasome/caspase-1/IL-1β signaling axis, independent of body weight and lipid profiles. More recently, orally bioavailable BAM15 has been reported to reverse diet-induced obesity and insulin resistance in mice, with strong antioxidant activities (*56*). These proof-of-concept studies, together with the ever-growing knowledge about uncoupling proteins, reignite interest in developing small-molecule mitochondrial uncouplers as a promising therapeutic solution to combat a myriad of diseases that are associated with mitochondrial dysfunction.

It has recently been reported that UCP1 antagonizes liver inflammation and pathology by reduction of circulating succinate (*25*). Succinate oxidation in UCP1+ brown adipocytes has been linked to ROS production, which is essential for thermogenesis in brown adipocytes (*26*). However, we found that pharmacological inhibition of SDH did not abolish the difference on mtSuperoxide accumulation between WT and UCP1-deficient PVAT, indicating that succinate is not a major culprit of inflammation within UCP1-deficient PVAT, possibly due to the fact that succinate uptake in adipocytes is low when UCP1 is absent (*25*). Together, these findings support the anti-inflammatory role of UCP1 through its direct action on PVAT to relieve high MMP-induced NLPR3 inflammasome activation as well as its indirect action on nonadipose tissues (such as the liver) by reducing excess amount of extracellular succinate (*25*).

Because of the lack of strategies to generate “PVAT-selective” KO mice at this stage, our study cannot exclude the possibility that the effects of UCP1 deficiency observed in vivo on exacerbation of vascular inflammation and atherosclerosis were partially attributed to its actions in other adipose depots other than PVAT. Nevertheless, we have provided evidence that UCP1 deficiency does not alter the inflammation factors either in the circulation or in BAT. Furthermore, reconstitution of UCP1 in iBAT did not reverse the exacerbated atherosclerosis in UCP1-deficient mice, indicating that the vascular modulation by UCP1 is at least partially attributed to UCP1 in PVAT. We also carried out ex vivo studies demonstrating that UCP1 in PVAT confers direct protection against endothelial dysfunction in intact aortic rings from mouse and pig models. Our coculture experiments showed that short-term treatment of PVAT with BAM15 or coincubation with IL-1β–neutralizing antibody improved endothelium-dependent relaxation in obese PVAT. Collectively, our study supports the notion that the vascular protective effects of UCP1 are mediated at least in part by its anti-inflammatory actions in PVAT.

## MATERIALS AND METHODS

### Animal use

All animal experiments were conducted in accordance with the Guidelines of HKU Animal Care and Use Committee (CULATR 4783-18) and Institute of Zoology, Chinese Academy of Sciences (IOZ20180061). *Ucp1* KO mice and *Apoe* KO mice on a C57BL/6J background were purchased from the Jackson Laboratory (Bar Harbor, MA). *Ucp1* and *Apoe* DKO mice were obtained by mating *Ucp1* KO mice with *Apoe* KO mice. Mice were housed in a controlled environment (12-hour light/dark cycle, 23°C ± 1°C, 60 to 70% humidity) and fed ad libitum with standard chow (LabDiet 5053, LabDiet), high-fat diet (D12451, Research Diets), or HFHC diet (D12108C, Research Diets). The metabolic parameters, including serum levels of triglyceride, total cholesterol, and high-density lipoprotein and low-density lipoprotein cholesterol, were measured as described (*57*).

For pig-related studies, Bama pigs with the mouse adiponectin gene promoter and *Ucp1* gene knocked in at the endogenous *UCP1* locus were constructed as reported (*28*). Generation of atherosclerosis pig model was carried out as described (*30*). Briefly, 6-month-old male UCP1 KI pigs and age- and sex-paired WT pigs were intravenously injected with streptozotocin for 3 days (130 mg kg^−1^ day^−1^) followed by HFHC diet (4% cholesterol + 17% saturated fat diet + 1% cholic acid) for 12 months. Age- and sex-matched WT pigs fed with normal diet were used as control. Body weight, plasma-fed glucose, and insulin levels were checked monthly.

### Assessment of endothelium-dependent vasorelaxation with wire myograph

The thoracic artery of the mice or LAD from pigs was isolated and transferred to an ice-cold modified Krebs solution (130 mM NaCl, 4.7 mM KCL, 1.19 mM KH_2_PO_4_, 1.18 mM MgSO_4_, 14.9 mM NaHCO_3_, 5.5 mM d-glucose, 1.8 mM CaCl_2_, and 30 μM EDTA, pH 7.4) aerated in 5% CO_2_:95% O_2_ gas mixture. Aortic rings (~2 mm) were mounted on a wire myograph (Danish Myo Technology, DMT) for continuous isometric tension measurement (AD Instruments Inc.). After equilibration for 25 min in modified Krebs at 37°C, the rings were stretched to 5 mN and contracted with 60 mM high-potassium solution (74.9 mM NaCl, 60 mM KCL, 1.19 mM KH_2_PO_4_, 1.18 mM MgSO_4_, 14.9 mM NaHCO_3_, 5.5 mM d-glucose, 1.8 mM CaCl_2_, and 30 μM EDTA, pH 7.4) to assess vessel vitality. The procedure was repeated three times. After washing, the rings were precontracted with U46619 (1 × 10^−8^ to 3 × 10^−8^ M), and endothelium-dependent relaxation was assessed by cumulative concentrations of the ACh from 10^−9^ to 10^−4^ M (for mice) and bradykinin from 10^−10^ to 10^−5^ M (for pig). ACh concentration responses were also conducted in the aortic ring after incubation with anti–IL-1β antibody (2 μg/ml; clone B122, eBioscience), the mitochondria-specific superoxide scavenger mito-TEMPO (5 μM; SML-0737, Sigma-Aldrich), or the mitochondrial uncoupler BAM15 (1 μM; st056388, TimTec, Newark, DE), 60 min before addition of U46619. Half-maximal relaxation (EC_50_) response to the concentration of ACh and maximal relaxation (*E*_max_) were calculated using GraphPad Prism software.

### Ultrasonography in mice

Ultrasound imaging was conducted using a Vevo 2100 high-frequency ultrasound scanner (Visualsonics Inc.) with an MS-550D linear array probe (40 MHz). Mice were anesthetized with isoflurane (1.5%) and positioned supine on a temperature- and electrocardiogram (ECG)–controlled board. The board temperature was maintained at 37°C. The chest and abdominal hair was removed, and ultrasound transmission gel was applied to acquire optimal image quality. Pulsed Doppler images were acquired at the thoracic and abdominal aorta sequentially. Pulse wave velocity was calculated by the distance between the thoracic and abdominal aortic sites divided by the time difference between the pulse arrivals. The time was measured from the R wave of the ECG to the foot of the blood flow waveform using digital calipers. The plaque burden of the aortic arch was observed by a long axis view on B-mode. The measurement site of plaques was at the origin of the brachiocephalic artery branch.

### Computed tomography in pigs

LADs of the pigs were scanned using a 64-slice Philips computed tomography (CT) scanner (Philips Medical Systems Ingenuity CT). After anesthesia, the pigs were centrally placed on a scanner table in prone position to ensure the entire heart was covered with the 22-cm field of view (FOV). Coronary angiography scan was initiated by continuously injecting a bolus of iohexol (600 mgI/kg, Tianheng Pharmacy) into an antecubital vein followed by saline solution (3 ml/s, 40 ml). Contrast medium application was controlled by the bolus-tracking method. A region of interest was placed into the aortic root, and image acquisition started 5 s after the signal intensity reached the predefined threshold of 100 Hounsfield units. Radiation dose reduction by ECG pulsing was used in all pigs. In each pig, images were reconstructed in 5% increments from 40 to 80% of R-R interval. All CT datasets were acquired with the following parameters: slice thickness, 0.90 mm; increment, 0.45 mm; and a medium soft-tissue convolution kernel (Xres Standard, XCB). The reconstructed FOV was adjusted to fully encompass the heart (image matrix, 512 × 512 pixels).

All reconstructed images were processed and analyzed by a cardiovascular postprocessing software (Comprehensive Cardiac Analysis). Coronary computed tomography angiography (CCTA) images were independently assessed by two experienced observers using multiplanar reformation, along with curve planar reformation and volume rendering.

### Injection of rAAV in iBAT

rAAV expressing mouse *Ucp1* or GFP (as control) in serotype 8 was prepared and purchased from Vector Builder. The injection procedure was performed as described (*58*). Briefly, 6-week-old male mice were anesthetized, shaved bilaterally to the spinal cord (roughly 1 cm below the head), and sterilized with three alternating wipes of Betadine and 70% ethanol. A shallow incision in the center of the shaved area (∼0.5 cm) was made to reveal the iBAT. Forceps were used to firmly grasp the BAT, and the rAAV was injected (1.0 × 10^9^ viral particles per 20 μl) with a 0.3-ml, 31-gauge insulin syringe. The tissue was lifted to ensure no virus leakage during injection. The procedure was repeated on the opposite side to complete the bilateral injection. The fat pad was gently forced back into the body cavity, the wound was closed with a 4-0 PDS II FS-2 suture, and the mice were allowed to recover in intensive care unit for 14 days before administration of high-cholesterol diet.

### Real-time polymerase chain reaction and Western blot analysis

Total RNA was extracted by RNAiso Plus (Takara) and reverse transcribed into complementary DNA using the PrimeScript Reverse Transcriptase (Takara). Real-time polymerase chain reactions (PCRs) were performed using SYBR Premix Ex Taq II (Takara) on a 7900 HT (Applied Biosystems), with the ribosomal protein *S*18 gene (*Rps18*) and *ACTIN* as a normalization control in mouse and pig samples, respectively. Primer sequences are listed in table S9. Proteins were extracted in RIPA buffer (0.5% NP-40, 0.1% sodium deoxycholate, 150 mM NaCl, and 50 mM tris-HCl, pH 7.4) containing complete protease inhibitor cocktail (Roche). The primary antibodies used are UCP1 (ab234430, Abcam), IL-1β (2022, Cell Signaling Technology), caspase-1 (AG-20B-0042, AdipoGen), and HSP90 (4874, Cell Signaling Technology). The protein bands were visualized with ECL Prime Western Blotting Detection Reagent (GE Healthcare) and quantified using the National Institutes of Health ImageJ software.

### Quantification of atherosclerotic plaques and immunostaining

The upper portion of the heart and proximal aorta of mice and the LADs of pigs were isolated and fixed in 4% paraformaldehyde overnight. Paraffin-embedded sections (5 μm) were deparaffinized and subjected to Masson trichrome staining (ab150686, Abcam). Double immunofluorescence staining was performed in aortic root sections of mice as described previously (*57*). Cryosections were immunostained with antibodies against MOMA-2 (1:100, Abcam) and α-smooth muscle actin (1:100, Sigma-Aldrich) overnight at 4°C. The sections were then washed and incubated for 1 hour at room temperature at dark with fluorochrome-conjugated secondary antibodies [Alexa Fluor 488 anti-rabbit, Alexa Fluor 488 anti-rat, or Alexa Fluor 555 anti-rat (1:1000) (Molecular Probes)]. Slides were counterstained with 4′,6-diamidino-2-phenylindole (DAPI). Mouse aortic root sections and the entire aorta trunk were stained by oil red O [60% (w/v)], and the plaque area was quantified by ImageJ software. For immunohistochemical staining, deparaffinized sections were incubated with affinity-purified rabbit antibody against UCP1 (ab234430, Abcam) or capthesin S (AF1183, R&D) in phosphate-buffered saline (PBS) containing 3% bovine serum albumin overnight at 4°C, followed by incubation with a horseradish peroxidase–conjugated secondary antibody against rabbit immunoglobulin G (IgG; Cell Signaling Technology) and developed by SIGMAFAST DAB (Sigma-Aldrich). Tissue sections were visualized with an Olympus biological microscope BX41, and images were captured with an Olympus DP72 color digital camera. The signal positive area was outlined manually and quantified by ImageJ software.

### Quantification of mitochondria superoxide and membrane potential

PVAT isolated from mice or pigs was immediately cryosectioned at 10 μm. The freshly sectioned tissues were incubated with mitoSox Red (5 μM; Thermo Fisher Scientific) or MitoTracker CMXRos (50 nM; Thermo Fisher Scientific) as specific dyes of mitochondria superoxide and mitochondria membrane potential, respectively, for 30 min at 37°C in high glucose–containing Dulbecco’s modified Eagle’s medium (DMEM). The tissues were then washed for three times in PBS and visualized with an Olympus biological microscope BX41, and images were captured with an Olympus DP72 digital camera. The relative intensity of the signal was quantified by ImageJ software. For each sample, 10 fields of view were used and averaged.

### Cell culture and NO production

HAoEC (PromoCell) were cultured in DMEM without phenol red containing l-glutamine (584 mg/liter) and fetal bovine serum (10%) (Thermo Fisher Scientific). The cells were seeded in 12-well plates at a density of 2 × 10^5^ cells per well and allowed to grow for another 2 days, followed by serum fasting overnight and subsequent stimulation with ACh (10 μM) in the absence or presence of recombinant human IL-1β (10 ng/ml; Sigma-Aldrich) for 15 min. For measurement of intracellular NO, 4,5-diaminofluorescein diacetate (DAF-2DA; 10 μM) (Sigma-Aldrich) was added to the cells for the last 5 min in the dark (37°C, 5 min). Fluorescence images were captured. Alternatively, HAoEC were seeded in 96-well plates at a density of 0.25 × 10^5^ cells per well and grown for 2 days. DAF-2DA (10 μM) was added 5 min before harvesting, and the fluorescence intensity of the cells was measured using a spectrofluorometer with excitation wavelength at 495 nm and emission wavelength at 515 nm. The relative fluorescence intensity was normalized with protein concentration in cell lysate.

### Measurement of IL-1β release from PVAT and blood vessels

PVAT and the blood vessels from mouse or pig aortas were separated and cultured in endothelial cell growth medium (C-22010, Promo Cell) for 16 hours. Alternatively, mouse PVAT was incubated with MCC950 (1 μM) for 16 hours or DMM (5 mM) for 4 hours before analysis. The conditioned medium was filtered through a 0.45-μm filter. IL-1β levels in mouse serum and conditioned medium were measured by mouse IL-1β enzyme-linked immunosorbent assay (ELISA) kit (Ab197742, Abcam). IL-1β in conditioned medium of pig PVAT was quantified by a sandwich immunoassay using anti-porcine IL-1β antibody (MAB6811, R&D, 2 μg/ml) and biotinylated anti-porcine IL-1β antibody (BAF681, R&D, 0.1 μg/ml) as a capture and detection antibody, and recombinant porcine IL-1β protein (681-PI, R&D) as the assay standard, respectively.

### Caspase-1 activity measurement

Caspase-1 activity was measured using caspase-1 colorimetric assay kit (K111-100, R&D). Briefly, cells and tissues were lysed in a cell lysis buffer containing 10 mM dithiothreitol. After centrifugation, lysates (100 μg) were mixed with YVAD-pNA substrate (200 μM) and incubated at 37°C for 1 hour, followed by reading at 405 nm in a microplate reader (BioTek).

### Statistical analysis

All analyses were performed with Statistical Package for Social Sciences version 14.0 (SPSS, Chicago, IL). Data were expressed as means ± SEM. For comparison of three or more experimental conditions, one-way analysis of variance (ANOVA) was applied for comparisons between multiple experimental groups, followed by post hoc analysis with the Fisher’s least significant difference. Student’s *t* test was used for comparison of two experimental conditions with normal distribution. Comparisons with *P* < 0.05 were considered statistically significant.

## Notes

View/request a protocol for this paper from *Bio-protocol*.
