# Supplementary material for: Mitochondrial uncoupling protein 1 antagonizes atherosclerosis by blocking NLRP3 inflammasome–dependent interleukin-1β production
Source: Sci Adv. 2021 Dec 8;7(50):eabl4024. doi: 10.1126/sciadv.abl4024 (PMC8654294; doi:10.1126/sciadv.abl4024)
Supplement: Supplementary file 1 — Figs. S1 to S12 Tables S1 to S9 [file sciadv.abl4024_sm.pdf]

Science Advances
AAAS

Supplementary Materials for

Mitochondrial uncoupling protein 1 antagonizes atherosclerosis by blocking NLRP3 inflammasome–dependent interleukin-1β production

Ping Gu, Xiaoyan Hui*, Qiantao Zheng, Yuan Gao, Leigang Jin, Weimin Jiang, Changsheng Zhou, Tianxia Liu, Yu Huang, Qing Liu, Tao Nie, Yanfang Wang, Yu Wang, Jianguo Zhao*, Aimin Xu*

*Corresponding author. Email: amxu@hku.hk (A.X.); hannahhui@cuhk.edu.hk (X.H.); zhaojg@ioz.ac.cn (J.Z.)

Published 8 December 2021, *Sci. Adv.* **7**, eabl4024 (2021)
DOI: 10.1126/sciadv.abl4024

This PDF file includes:

Figs. S1 to S12
Tables S1 to S9

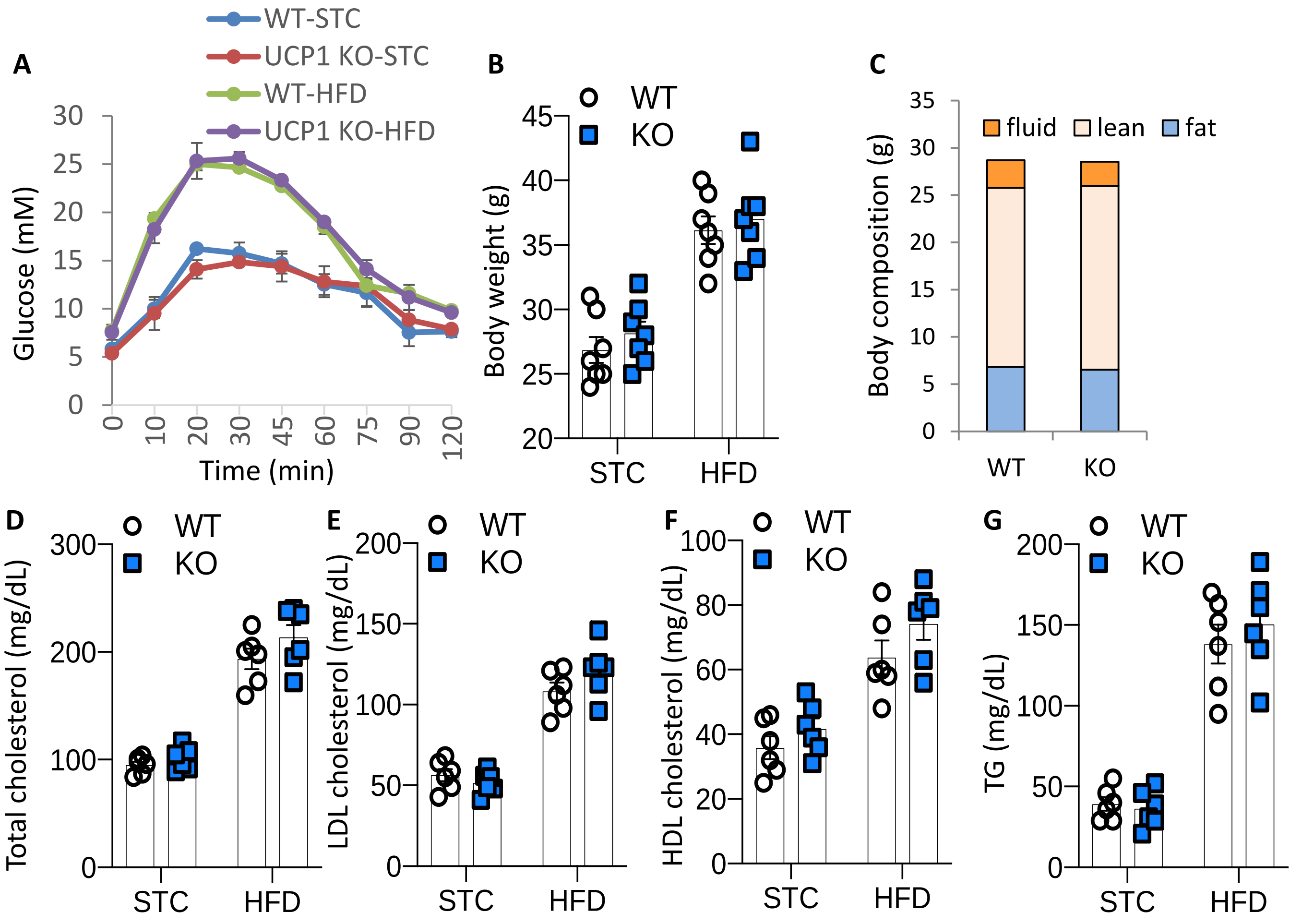

Fig. S1. UCP1 deficiency does not affect body weight, body composition, glucose and lipid metabolism. 8-week old male WT and *Ucp1* KO mice were fed with standard chow (STC) or high fat diet (HFD) for 12 weeks. (A) Glucose tolerance test. (B) body weight and (C) body composition of the mice at 12 weeks after feeding with STC or HFD. N=7. (D)-(G) Fasting lipid levels in mouse serum. LDL, low density lipoprotein; HDL, high density lipoprotein; TG, triglyceride. N=6.

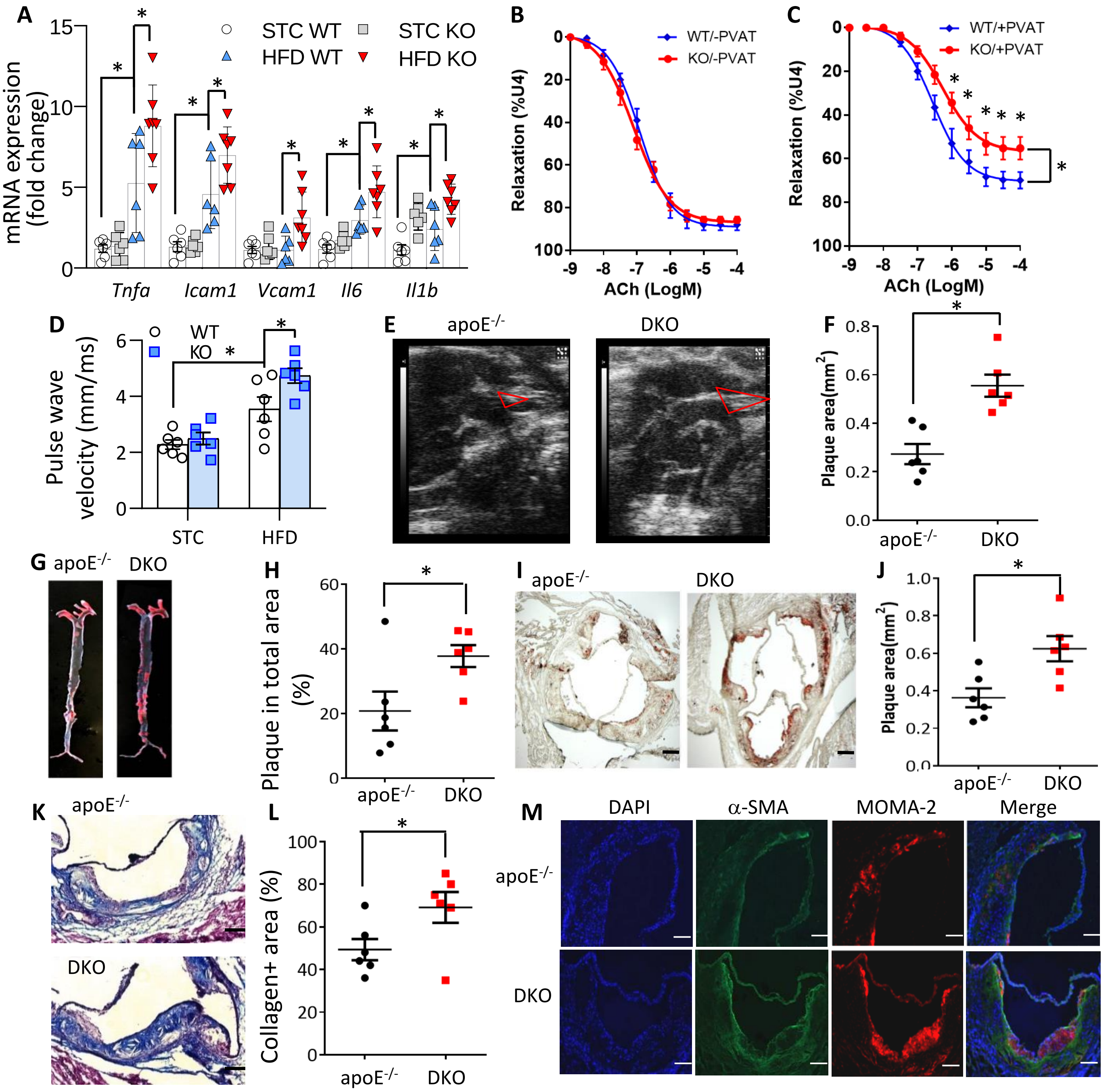

Fig. S2. *Ucp1* knockout mice are more susceptible to high fat diet-induced vascular inflammation, endothelial dysfunction and atherosclerosis. (A)-(D) 8-week-old male *Ucp1* knockout mice (KO) and their wildtype littermates (WT) were fed with STC or HFD for 12 weeks before analysis. (A) Real time PCR analysis for mRNA expressions of inflammation markers in mouse aorta. N=6-7. (B)-(C) ACh-evoked vasorelaxation in aortic rings without (B) or with (C) PVAT measured by myograph. N=8-9. (D) Pulse wave velocity in thoracic aorta of the mice. N=6. (E)-(L) 8-week-old male *Ucp1*, apoE double knockout (DKO) and apoE knockout mice (apoE$^{-/-}$) were fed with high fat high cholesterol (HFHC) diet for 12 weeks. (E) Visualization of aortic arch by ultrasonography. The plaque at the brachiocephalic artery branch is illustrated in triangle. (F) Plaque area at the brachiocephalic artery as quantified by Vevo Lab software. N=6. (G) Representative photographs of aortas in the *en face* preparation after staining with oil red O. (H) Percentage of oil red O-stained plaque area in entire aorta. (I) Representative images of the aortic sinus stained with oil red O (Scale bar: 200μm) and (J) quantification of atherosclerotic area. N=6. (K) Representative images of Masson trichrome-staining in aortic root. Scale bar: 100μm. (L) Collagen-positive area in the lesion of the aortic sinus. N=6. (M) Immunohistological analysis of atherosclerotic lesion areas in aortic sinuses with antibodies against α-smooth muscle actin (α-SMA) and MOMA-2. Scale bar: 100μm. *p<0.05. Photo Credit: Xiaoyan Hui, The University of Hong Kong.

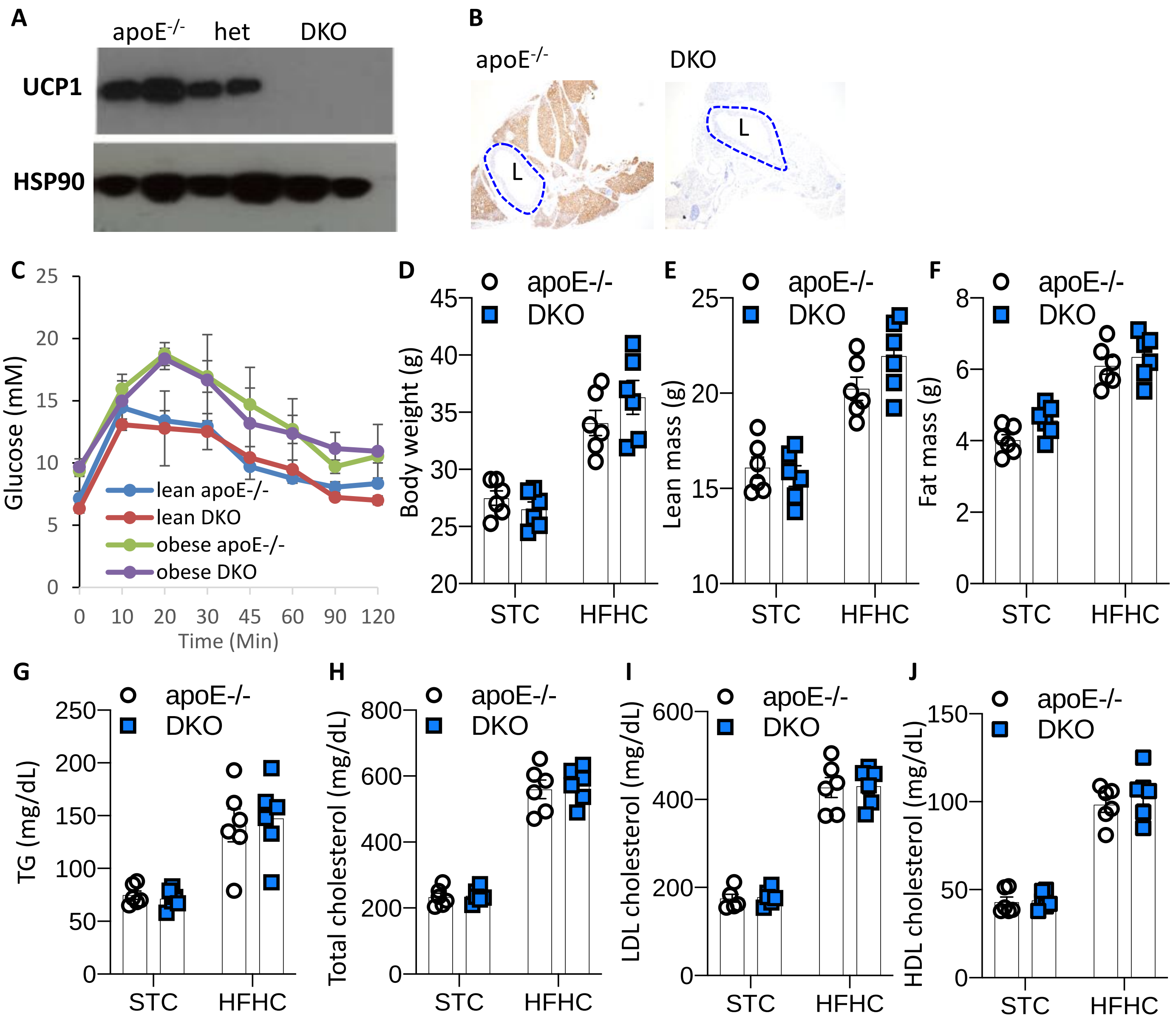

Fig. S3. Metabolic parameters of apoE$^{-/-}$ and DKO mice. Ucp1 KO mice were mated with apoE$^{-/-}$ mice to generate Ucp1/apoE double knockout mice (DKO). (A) Western blot analysis of UCP1 in PVAT of apoE$^{-/-}$, *Ucp1* heterogenous (het) and homogeneous (DKO) mice. (B) Immunohistochemical staining of UCP1 in mouse aorta with PVAT. The blue dotted line delineates the blood vessel. L, lumen. (C)-(J) apoE$^{-/-}$ and DKO mice were fed with standard chow (STC) or high fat high cholesterol diet (HFHC) for 12 weeks. (C) Glucose tolerance test; (D)-(F) Body weight and body composition of the mice at 12 weeks after feeding STC or HFD. (G)-(J) Fasting lipid levels (TG, total, LDL and HDL cholesterol) in serum. LDL, low density lipoprotein; HDL, high density lipoprotein; TG, triglyceride. N=6.

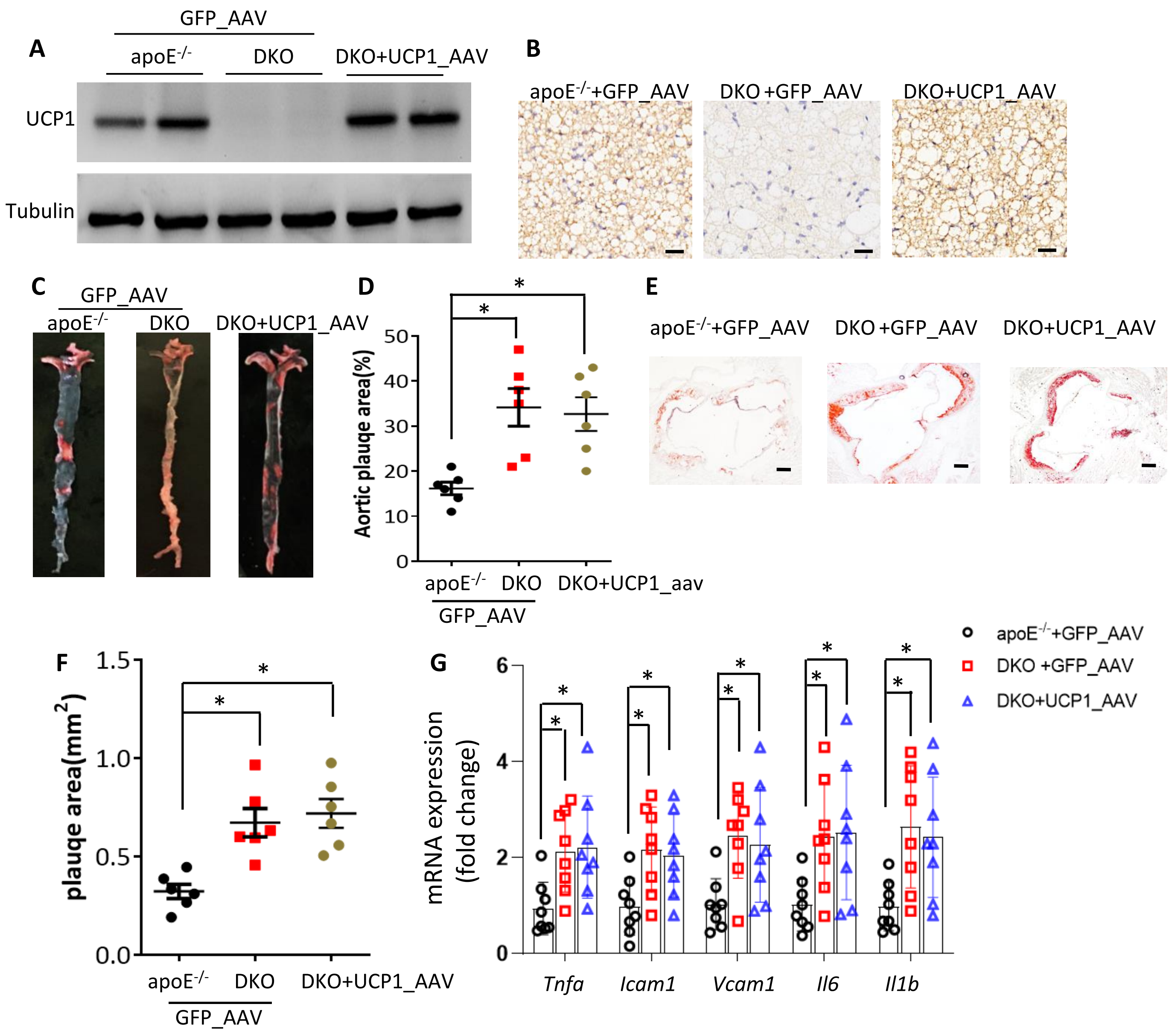

Fig. S4. Reconstitution of UCP1 in BAT does not rescue the exacerbated vascular inflammation and atherosclerosis in DKO mice. 6-week-old *Ucp1*, apoE double knockout (DKO) and apoE knockout mice (apoE$^{-/-}$) were injected with recombinant adeno associated virus (AAV) expressing UCP1 or GFP at the interscapular BAT. After two weeks, the mice were fed with high fat high cholesterol (HFHC) diet for 12 weeks before analysis. (A) Western blot and (B) Immunohistochemical staining of UCP1 in mouse BAT. (C) Representative photographs of aortas in the en face preparation after staining with oil red O. (D) Percentage of oil red O-stained plaque area in entire aorta. (E) Representative images of the aortic sinus stained with oil red O (Scale bar: 200µm) and (F) quantification of atherosclerotic area. N=6. (G) The mRNA expressions of pro-inflammatory genes in mouse aorta. N=8. *p<0.05. Photo Credit: Xiaoyan Hui, The University of Hong Kong.

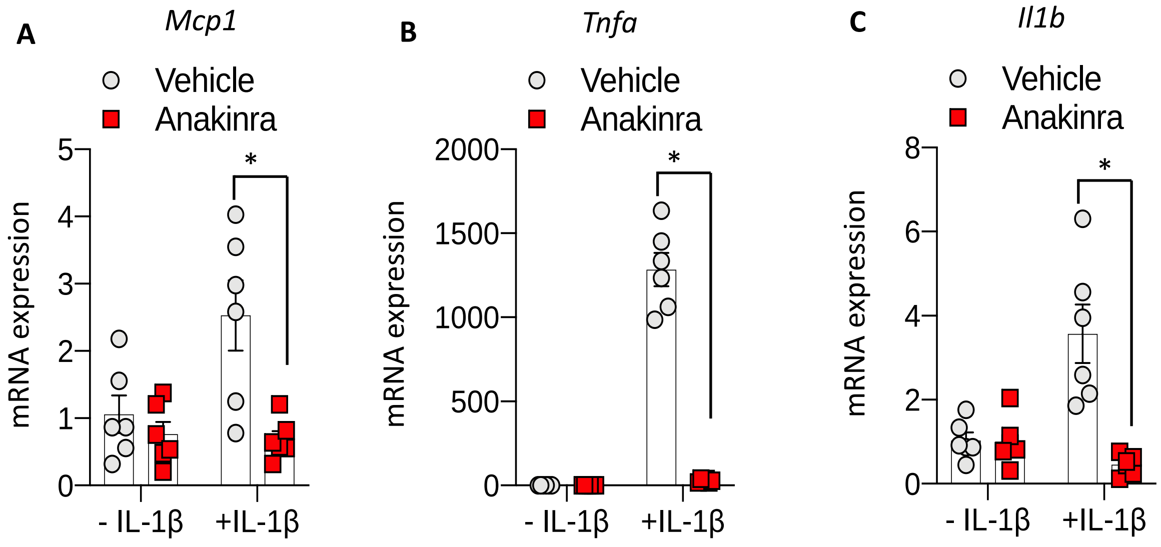

Fig. S5. Validation of the inhibitory effect of Anakinra on IL-1β-induced inflammation. Bone marrow cells were isolated from C57BL/6J mice and differentiated in vitro into macrophages. Cells were pretreated with Anakinra (5ng/ml) or PBS (Vehicle) for 1 hour, followed by addition of recombinant human interleukin 1β (10ng/ml). The cells were harvested 24 hours after treatment and subjected to real time PCR analysis for mRNA expression of several inflammatory genes. N=6. *$p<0.05$.

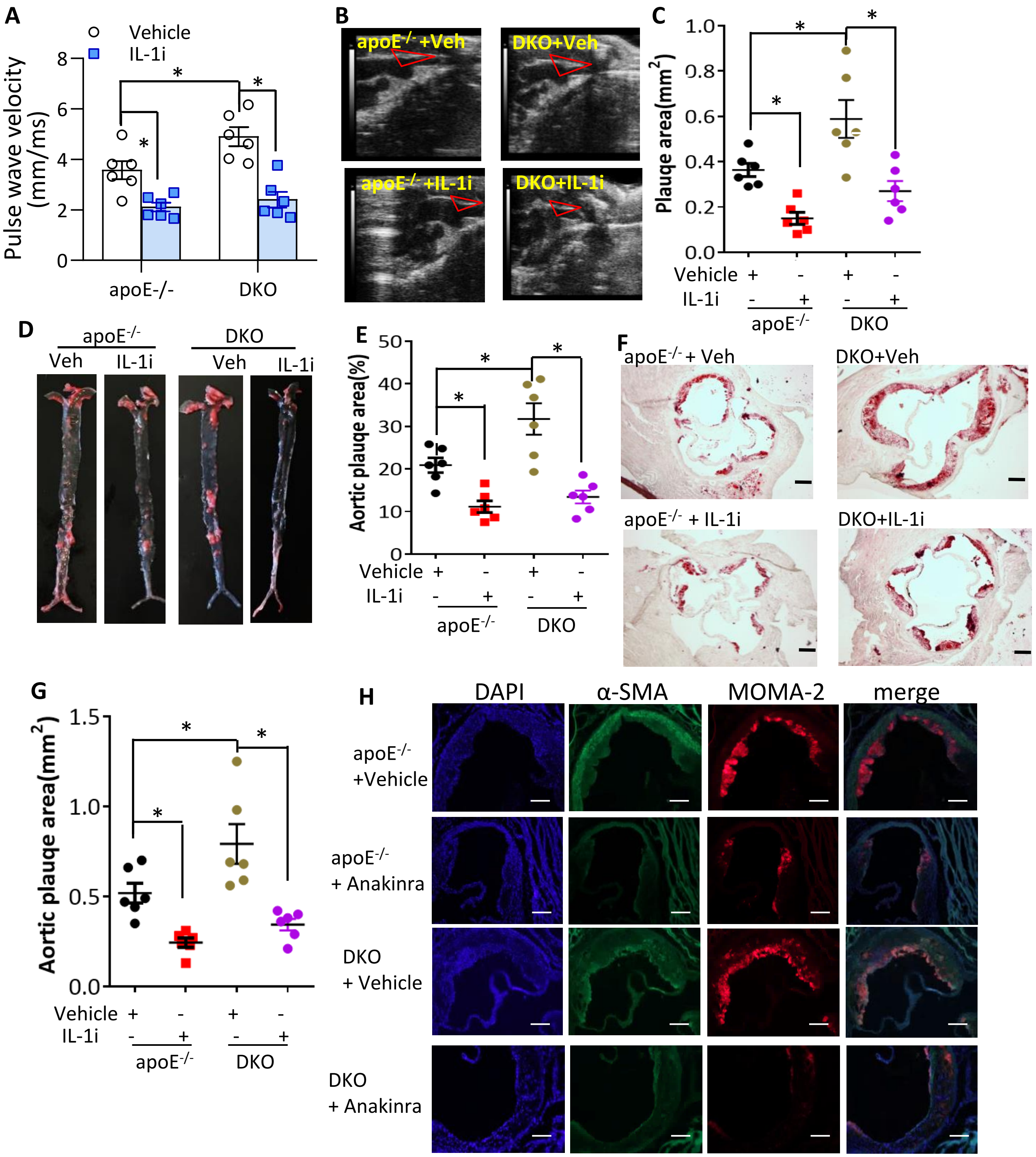

Fig. S6. Anakinra decreases UCP1 deficiency-induced exacerbation of vascular inflammation, endothelial dysfunction and atherosclerosis in mice. HFHC diet-fed DKO and apoE$^{-/-}$ mice were treated with the IL-1R agonist Anakinra (IL-1i, 10mg/kg/d) or PBS as vehicle (Veh) by daily intraperitoneal injection for 12 weeks. (A) Ultrasonic measurement of local aortic pulse wave velocity. N=6. (B) Visualization of the brachiocephalic artery branches by ultrasonography. Triangle illustrates the observed plaques at the brachiocephalic branch. N=6. (C) Quantification of the plaque areas visualized with ultrasonography. (D) Representative microphotographs of en face aortas stained with oil red O and (E) quantification of the plaque area. N=6. (F) Representative images oil red O-stained aortic sinus. Scale bar: 200µm. (G) Quantification of the lesion area in aortic sinus. N=6. (H) aorta roots were subjected to Immunofluorescent staining for MOMA-2 and α-actin, respectively. Scale bar: 100µm. *$p<0.05$.

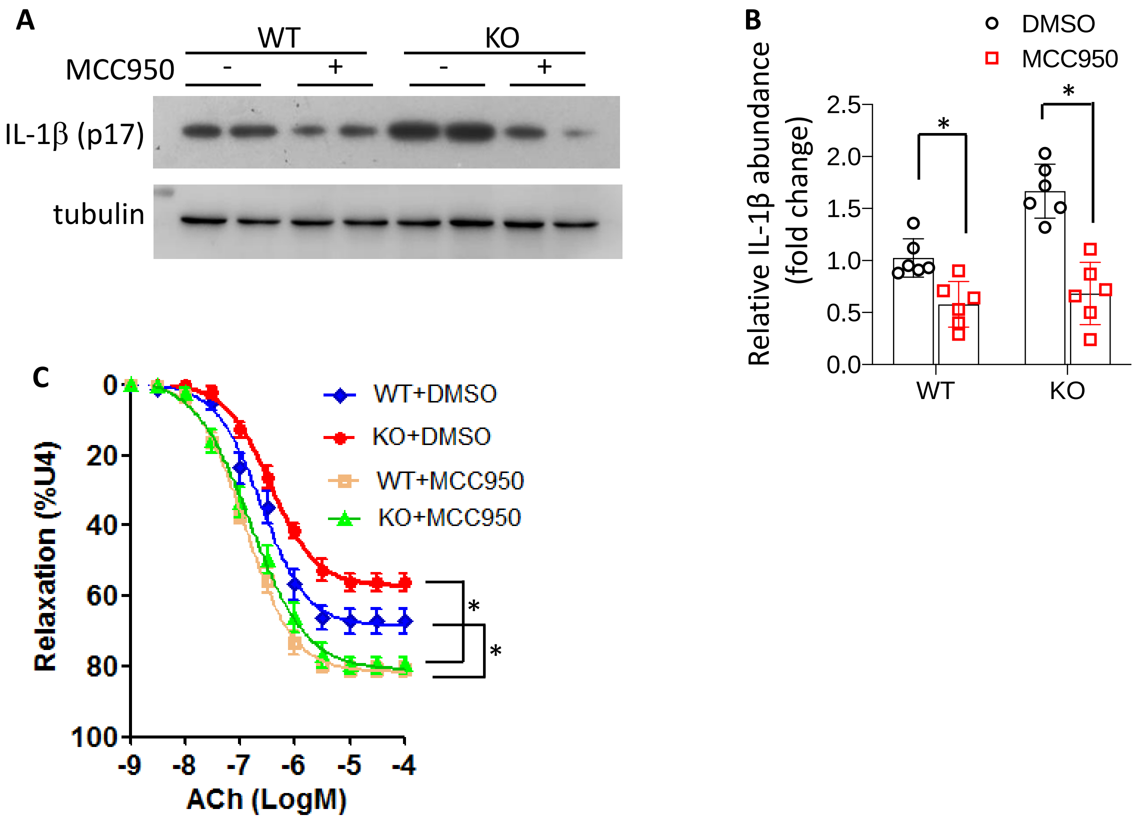

Fig. S7. Inhibition of NLPR3 inflammasome reverses UCP1 deficiency-induced augmentation of IL-1β maturation in PVAT and UCP1-deficient PVAT- endothelial dysfunction of mouse aortas. PVAT was isolated from WT and Ucp1 KO mice on HFD for 8 weeks, incubated with MCC950 (1μM) or DMSO for 16 hours and was then co-incubated with the aortic rings from lean WT mice. (A) Western blot analysis for the level of mature IL-1β in PVAT. (B) Densitometry quantification of IL-1β (p17) band in (A), normalized with β-tubulin. N=6, *$p<0.05$. (C) ACh-induced vasorelaxation in mouse aortic rings cocultured with PVAT pretreated with MCC950 or DMSO. N=7-8. *$p<0.05$ vs. +MCC950.

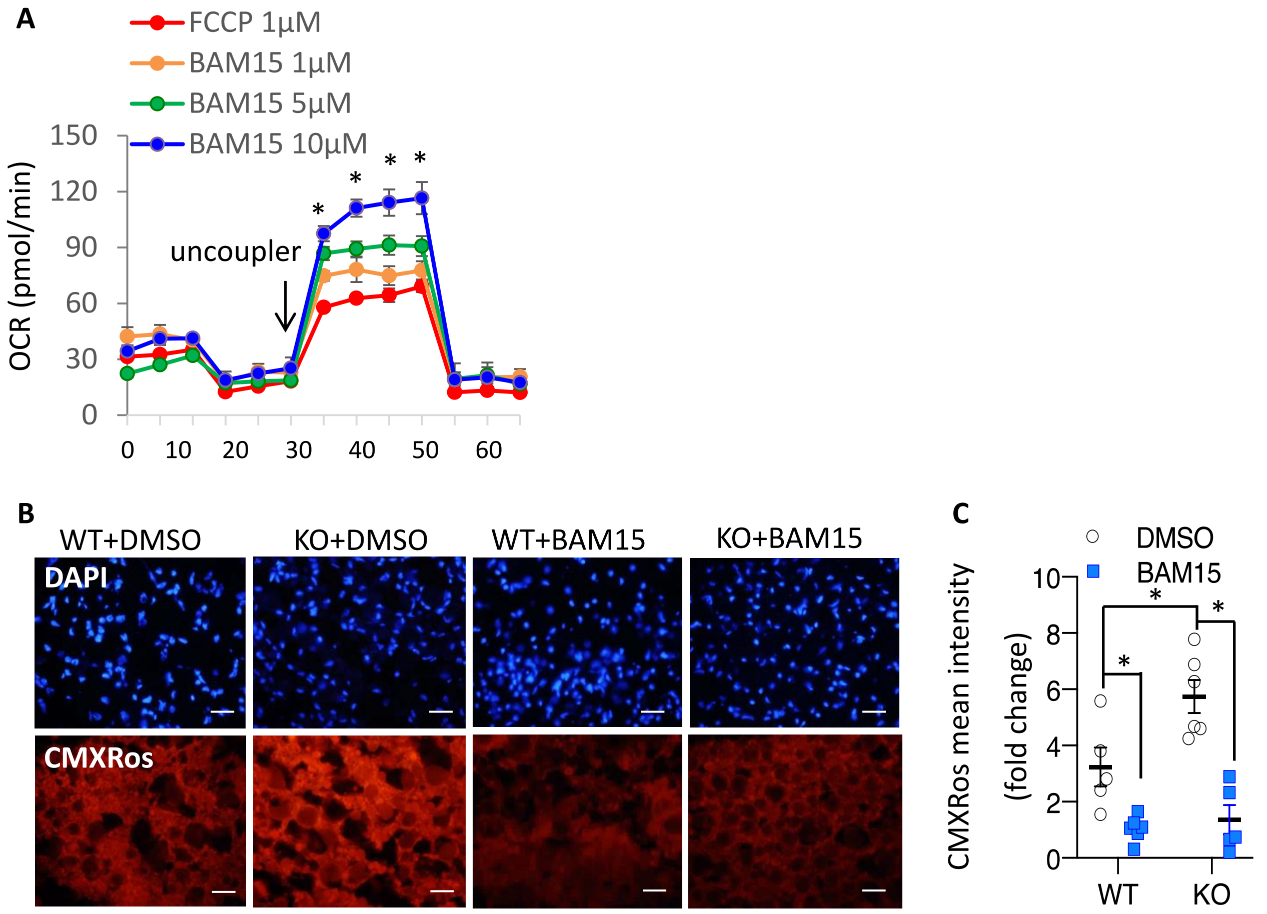

Fig. S8. BAM15 reduces MMP *in vitro* and *ex vivo*. (A) Adipocytes differentiated from stromal vascular fraction of subcutaneous white adipose tissue from 8-week-old male C57BL/6J mice were subjected to measurement of oxygen consumption rate (OCR) with seahorse XF analyzer. The uncoupling ability of mitochondria uncoupler (FCCP) or BAM15 at different concentrations was tested. *p<0.05 vs. FCCP 1µM. (B) PVAT from WT and *Ucp1* KO mice were preincubated with BAM15 or vehicle (DMSO) before staining with CMXRos. Scale bar: 20µm. (C) Quantification of fluorescence intensity in (B). N=5-6. *p<0.05.

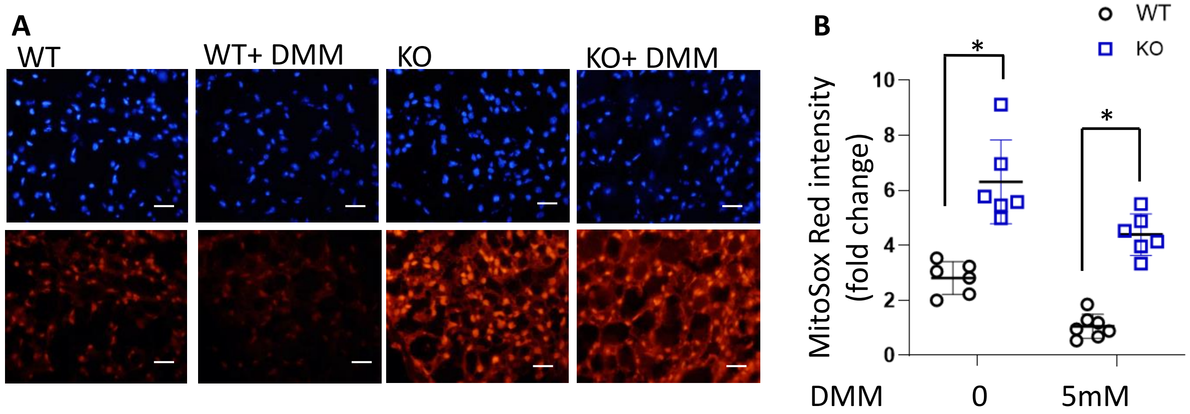

Fig. S9. Pharmacological inhibition of succinate dehydrogenase does not alter UCP1 deficiency-induced elevation of mtSuperoxide production in PVAT. PVAT was isolated from male WT and Ucp1 KO mice on HFD for 8 weeks and incubated with dimethyl malonate (DMM, 5mM) for 4 hr before staining with MitoSox Red. Scale bar: 20µm. (B) Quantification of fluorescence intensity in (A). N=6. *p<0.05.

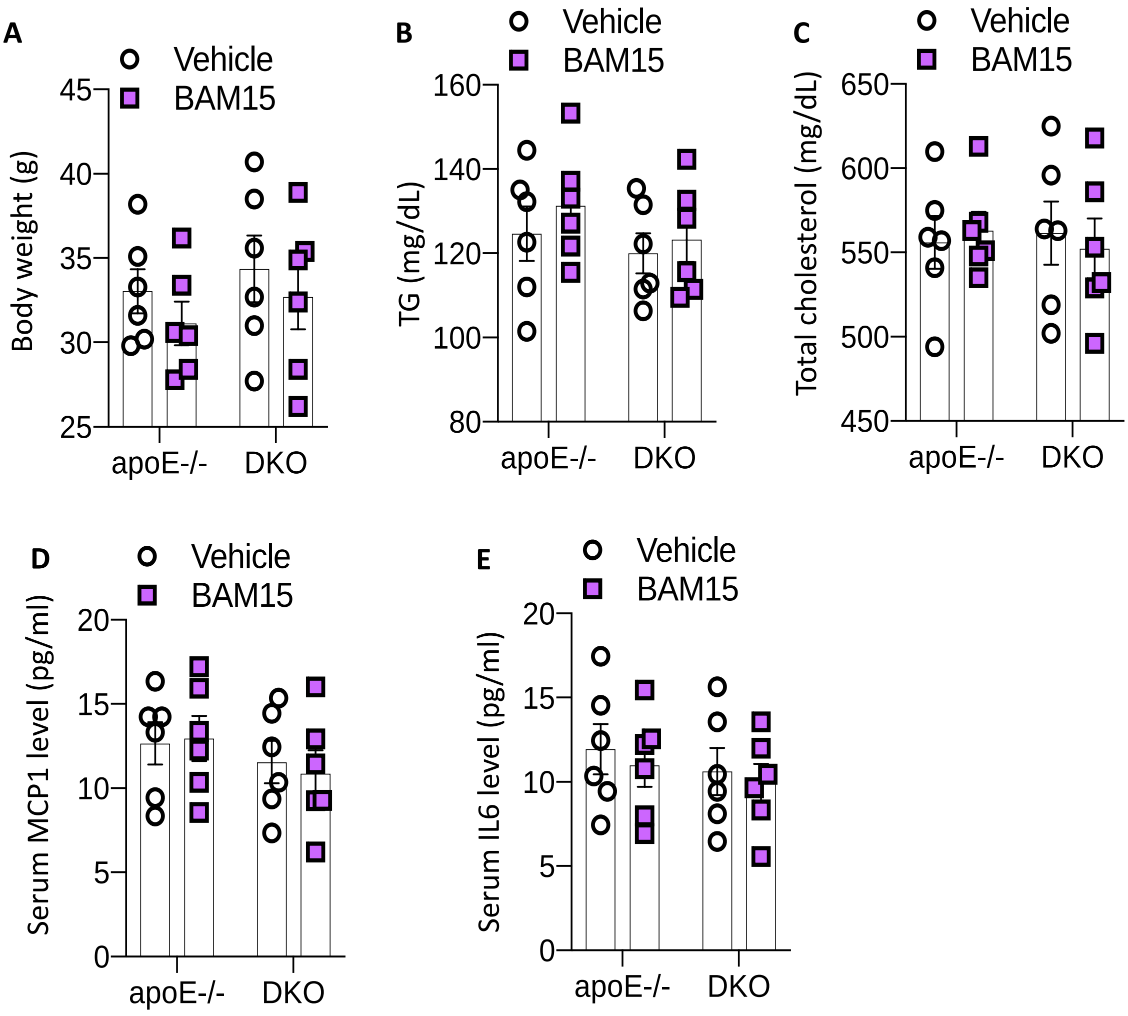

Fig. S10. The mitochondrial uncoupler BAM15 does not influence body weight, lipid metabolism and circulating inflammatory cytokines in apoE$^{-/-}$ and DKO mice. DKO and apoE$^{-/-}$ mice were fed with HFHC diet and were treated with of BAM15 (0.5mg/kg/d) or vehicle control by daily intraperitoneal injection for 12 weeks. Body weight (A), serum TG (B) and total cholesterol level (C) of the mice. Serum MCP1 (D) and IL-6 (E) in mice. N=6.

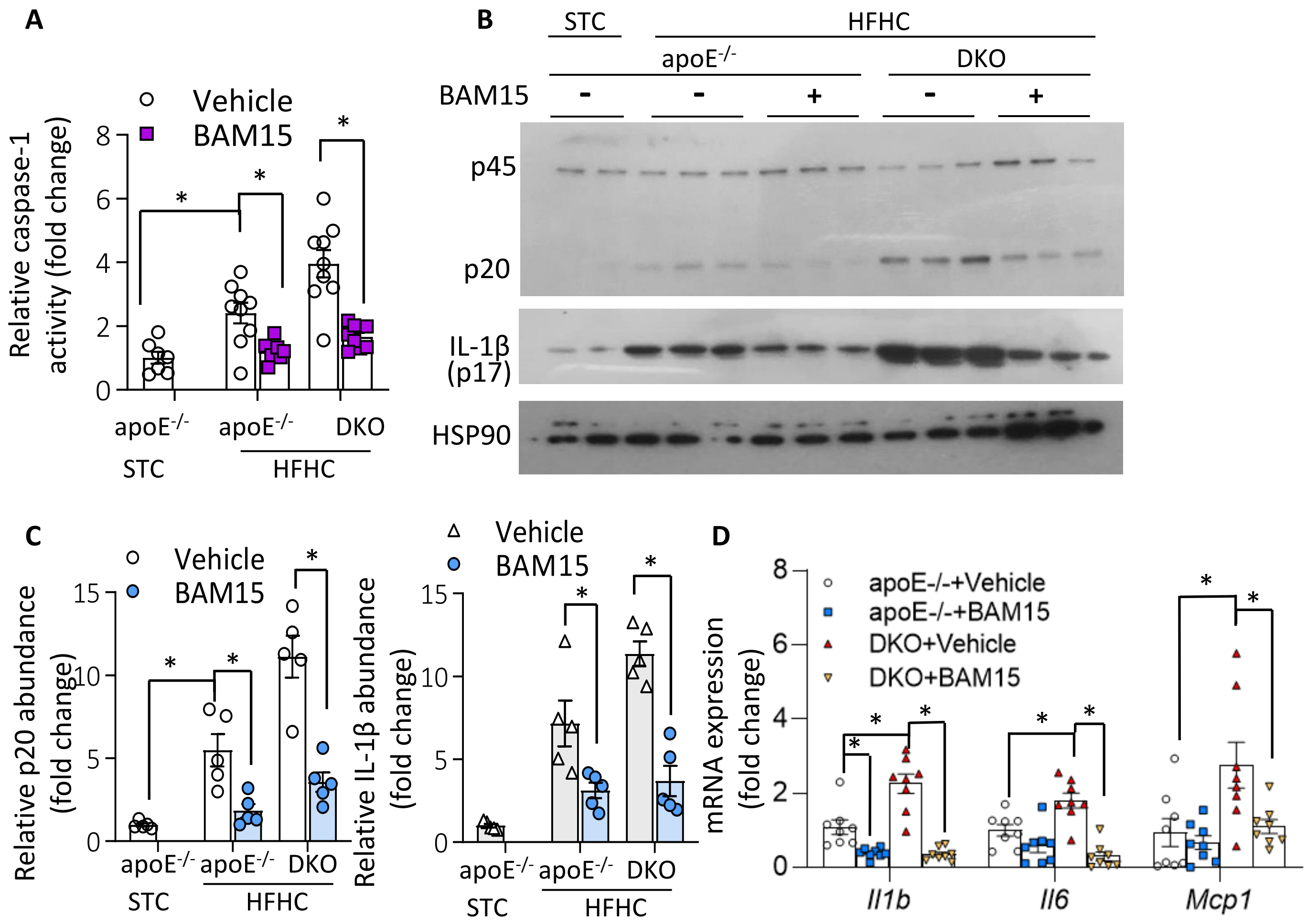

Fig. S11. BAM15 inhibits UCP1 deficiency-induced inflammasome activation and IL-1β in PVAT. DKO and apoE$^{-/-}$ mice on standard chow (STC) or HFHC diet were treated with BAM15 (0.5mg/kg/d) or vehicle control (3%DMSO 50% PEG-400) by daily intraperitoneal injection for 8 weeks. After sacrificing the mice, aortas were collected to separate PVAT and vessel wall for further analysis. (A) Biochemical analysis for Caspase-1 activity in PVAT. N=7-9. (B) Western blot analysis for the abundance of procaspase-1 (p45), mature form of caspase-1 (p20) and IL-1β (p17) in PVAT. (C) Densitometric quantification for mature form of caspase-1 and IL-1β. N=5. (D) Real time PCR analysis for mRNA expression of several pro- inflammatory genes in mouse aorta. N=8. *$p<0.05$.

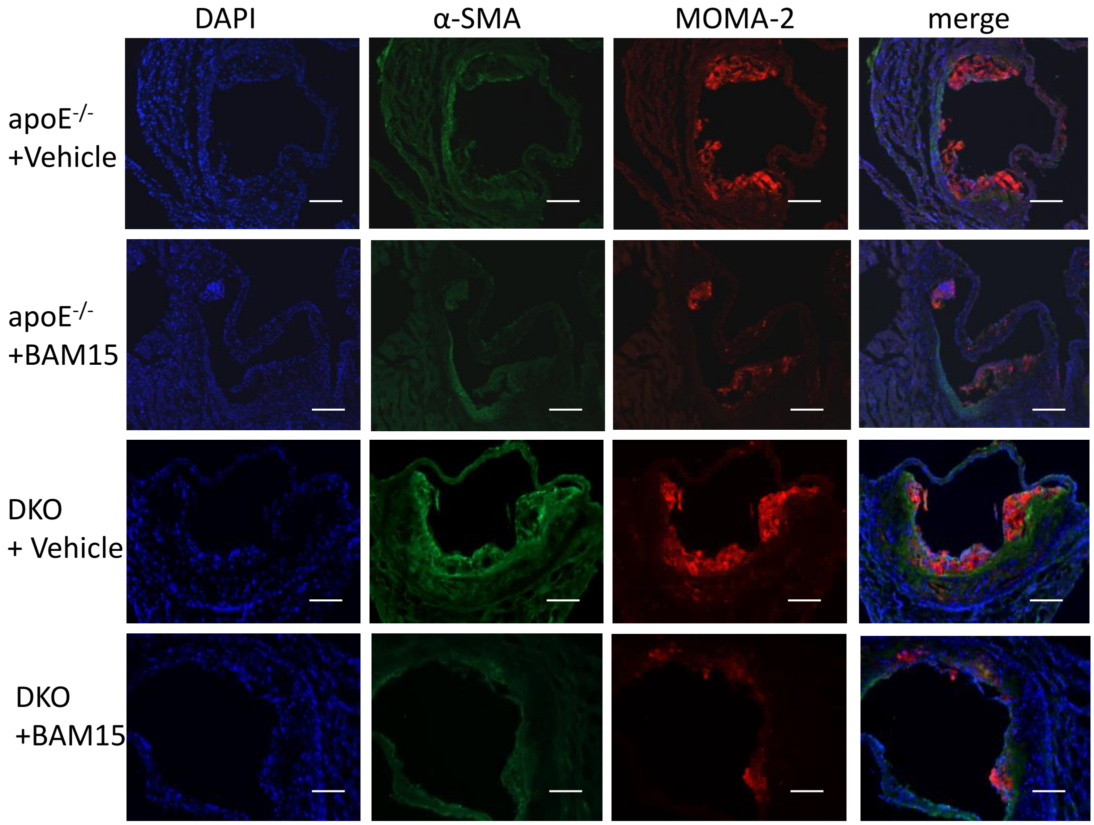

Fig. S12. BAM15 alleviates UCP1 deficiency-induced exacerbation of smooth muscle proliferation and macrophage infiltration in atherosclerotic lesions of apoE$^{-/-}$mice. DKO and apoE$^{-/-}$ mice on HFHC diet were treated with BAM15 (0.5mg/kg/d) or vehicle for 12 weeks as in Figure 7. After sacrificing the mice, the sections of aortic tissues were subjected to Immunofluorescence staining for DAPI, MOMA-2 and α-smooth muscle actin, respectively. Scale bar: 100μm.

		Emax		LogEC50	
		STC	HFD	STC	HFD
-PVAT	Mean (SD)	87.75 (7.89)	85.14 (15.52)	-7.02 (0.21)	-6.74 (0.83)
+PVAT	Mean (SD)	87.63 (7.74)	64.50 (13.60)* #	-6.26 (0.41) #	-5.27 (0.42)* #

Table S1. Effects of PVAT on potency and efficacy of endothelium-dependent relaxations in aorta from lean and obese mice. 8-week-old male C57BL/6J mice were fed with standard chow (STC) or high fat diet (HFD) for 12 weeks. The maximal relaxation (Emax) and EC50 in response to acetylcholine (ACh) in aortic rings without (-PVAT) or with (+PVAT) were determined by wire myograph. * $p<0.05$ vs. STC; # $p<0.05$ vs. –PVAT. N=7-8.

		Emax		LogEC50	
		24°C	6°C	24°C	6°C
-PVAT	Mean (SD)	84.91 (8.58)	87.63 (6.96)	-6.85 (0.44)	-7.10 (0.60)
+PVAT	Mean (SD)	65.25 (7.30)*	74.59 (7.36) * #	-5.50 (0.40)*	-5.96 (0.31)* #

Table S2. Effects of cold challenge on potency and efficacy of endothelium-dependent relaxations in mouse aorta. C57BL/6J mice on HFD for 12 weeks were housed at 6°C or room temperature (24°C) for 6 days. The Emax and EC50 of ACh in aortic rings with (+PVAT) or without PVAT (-PVAT) were determined by wire myograph. * p<0.05 vs. -PVAT; # p<0.05 vs. 24°C. N=13-15.

		Emax		LogEC50	
		**WT**	**KO**	**WT**	**KO**
**-PVAT**	**Mean (SD)**	**87.97 (7.30)**	**85.67 (4.11)**	**-6.67 (0.42)**	**-6.78 (0.34)**
**+PVAT**	**Mean (SD)**	**69.87 (11.19)***	**55.17 (15.29)* #**	**-5.68 (0.62)***	**-4.74 (0.92)* #**

Table S3. Potency and efficacy of endothelium-dependent relaxation in aorta of *Ucp1* KO and WT mice. 8-week-old male *Ucp1* knockout mice (KO) and their wildtype littermates (WT) were fed with STC or HFD for 12 weeks. The Emax and EC50 of ACh in aortic rings without (-PVAT) or with (+PVAT) were measured by wire myograph. * $p<0.05$ vs. WT; # $p<0.05$ vs. –PVAT. N=8-9.

		Emax		LogEC50	
		WT	KO	WT	KO
Control IgG	Mean (SD)	63.12 (2.20)	50.56 (5.39)*	-5.35 (0.16)	-4.40 (0.36)*
Anti-IL-1β	Mean (SD)	76.88 (11.19)#	70.15 (6.96) #	-6.05 (0.65)#	-5.59 (0.40)#

Table S4. Effects of IL-1β neutralizing antibody on potency and efficacy of endothelium-dependent relaxation in mouse aorta. WT and *Ucp1* KO mice were fed on HFD for 12 weeks and the aortic rings with PVAT were isolated and incubated with hamster anti-IL-1β IgG or isotype-matched control IgG (2 μg/ml) for 1 h. After that, The Emax and EC50 of ACh were quantified by wire myograph. * $p<0.05$ vs. aorta from WT mice; # $p<0.05$ vs. control IgG. N=6-7.

		Emax		LogEC50	
		WT	KO	WT	KO
control	Mean(SD)	67.27(9.27)	56.25(5.08)*	-5.69(0.49)	-5.08(0.39)*
MCC950	Mean(SD)	80.84(5.80)#	79.80(6.77)#	-6.47(0.30)#	-6.31(0.39)#

Table S5. Effects of MCC950 on potency and efficacy of endothelium-dependent relaxation in Ucp1 KO and WT mice. WT and Ucp1 KO mice fed on HFD for 12 weeks before PVAT was isolated and treated ex vivo MCC950 (1 μM) or DMSO. Aortic rings from 8-week-old lean C57BL/6J mice were mounted on wire myograph machine and co-incubated with pre-treated PVAT in chamber. The Emax and EC50 of ACh were then determined by wire myograph. * $p<0.05$ vs. WT; # $p<0.05$ vs. control. N=7-8.

		Emax		LogEC50	
		WT	KO	WT	KO
Vehicle	Mean(SD)	68.39(11.79)	55.75(15.82)*	-5.73 (0.62)	-4.94(0.98)*
mito-TEMPO	Mean(SD)	82.03(6.96)#	78.55(13.03) #	-6.43(0.30)#	-6.24(0.67)#

Table S6. Effects of the mitochondrial superoxide chelator mito-TEMPO on potency and efficacy of endothelium-dependent relaxation. WT and *Ucp1* KO mice were fed on HFD for 12 weeks. The intact aortic rings were pre-treated with Mito-TEMPO or vehicle (DMSO) for 10 min, followed by measurement of the Emax and EC50 of ACh by wire myograph. *p<0.05 vs. aorta from WT; # p<0.05 vs. vehicle. N=15.

		Emax		LogEC50	
		WT	KO	WT	KO
control	Mean(SD)	69.25(3.75)	59.78(5.08)*	-5.79(0.30)	-5.25(0.31)*
BAM15	Mean(SD)	85.19(3.95)#	81.13(3.04)#	-6.43(0.25)#	-6.33(0.12)#

Table S7. Effects of BAM15 on potency and efficacy of endothelium-dependent relaxation in *Ucp1* KO and WT mice. WT and *Ucp1* KO mice fed on HFD for 12 weeks before PVAT was isolated and treated ex vivo with BAM15 (1 µM) or DMSO for 1 h. Aortic rings from 8-week-old lean C57BL/6J mice were mounted on wire myograph machine and co-incubated with pre-treated PVAT in chamber. The Emax and EC50 of ACh were then determined by wire myograph. * $p<0.05$ vs. WT; # $p<0.05$ vs. control. N=6-7.

		Emax			LogEC50		
		ND WT	DMHC WT	DMHC KI	ND WT	DMHC WT	DMHC KI
-PVAT	Mean (SD)	96.57 (2.08)	75.20 (10.08)*	76.36 (8.40)*	-8.33 (0.47)	-7.09 (0.37)*	-7.04 (0.55)*
+PVAT	Mean (SD)	78.01 (3.38)△	48.84 (15.36)* △	73.23 (5.51)#	-7.04 (0.46) △	-5.40 (0.86)* △	-6.77 (0.35)#

Table S8. Potency and efficacy of endothelium-dependent relaxation in the coronary arteries of WT and UCP1 KI pigs. WT and UCP1 knockin (KI) pigs were induced to diabetes by injection of streptozotocin (STZ), followed by high fat high cholesterol feeding (DMHC) for 12 months. Age-paired WT pigs fed with normal diet (ND) were used as the control. The Emax and EC50 of bradykinin in LAD rings from ND WT pig and DMHC pigs were measured by wire myograph. *p<0.05 vs. NDWT, # p<0.05 vs. DMHC WT, △p<0.05 vs. -PVAT. N=6.

Name	Forward (5' – 3')	Reverse (5' – 3')	Species
Mcp1	CATCCACGTGTTGGCTCA	GATCATCTTGCTGGTGAATGA	Mouse
Tnf-α	ACGGCATGGATCTCAAAGAC	AGATAGCAAATCGGCTGACG	Mouse
Il-1b	GCAACTGTTCCTGAACTCAACT	ATCTTTTGGGGTCCGTCAACT	Mouse
Rps18	AGTTCCAGCACATTTTGCGAG	TCATCCTCCGTGAGTTCTCCA	Mouse
Icam1	GTGATGCTCAGGTATCCATCCA	CACAGTTCTCAAAGCACAGCG	Mouse
Vcam1	AGTTGGGGATTCGGTTGTTCT	CCCCTCATTCCTTACCACCC	Mouse
Il6	CTCTGGGAAATCGTGGAAATG	AAGTGCATCATCGTTGTTCATACA	Mouse
Adpn	GGAGAGAAAGGAGATGCAGGT	CTTTCCTGCCAGGGGTTC	Mouse
Leptin	CAAGCAGTGCCTATCCAGA	AAGCCCAGGAATGAAGTCCA	Mouse
TNFA	CTCTTCTGCCTACTGCACTTCG	GCTTTGACATTGGCTACAACG	Pig
VCAM1	CCACGCTGGTCATGAATCCCGT	AACGTCAGGCACCAGACACCTGA	Pig
ICANM1	CAGTGTTGCCTGTGATGGAAA	CTTCAGTCTTGTGCCAGTGAGTCT	Pig
ACTIN	CTGGGGCCTAACGTTTCTCAC	GTCCTTTCTTCCCCGATGTT	Pig

Table S9. Sequence of the primers used in the study.
